# Interactions between Type 1 Interferons and the Th17 Response in Tuberculosis: Lessons Learned from Autoimmune Diseases

**DOI:** 10.3389/fimmu.2017.00294

**Published:** 2017-04-05

**Authors:** Bas C. Mourik, Erik Lubberts, Jurriaan E. M. de Steenwinkel, Tom H. M. Ottenhoff, Pieter J. M. Leenen

**Affiliations:** ^1^Department of Medical Microbiology and Infectious Diseases, Erasmus University Medical Center, Rotterdam, Netherlands; ^2^Department of Rheumatology, Erasmus University Medical Center, Rotterdam, Netherlands; ^3^Department of Infectious Diseases, Leiden University Medical Center, Leiden, Netherlands; ^4^Department of Immunology, Erasmus University Medical Center, Rotterdam, Netherlands

**Keywords:** *Mycobacterium tuberculosis*, autoimmune diseases, neutrophils, inflammation, tertiary lymphoid structures, antibodies, B-cell-activating factor

## Abstract

The classical paradigm of tuberculosis (TB) immunity, with a central protective role for Th1 responses and IFN-γ-stimulated cellular responses, has been challenged by unsatisfactory results of vaccine strategies aimed at enhancing Th1 immunity. Moreover, preclinical TB models have shown that increasing IFN-γ responses in the lungs is more damaging to the host than to the pathogen. Type 1 interferon signaling and altered Th17 responses have also been associated with active TB, but their functional roles in TB pathogenesis remain to be established. These two host responses have been studied in more detail in autoimmune diseases (AID) and show functional interactions that are of potential interest in TB immunity. In this review, we first identify the role of type 1 interferons and Th17 immunity in TB, followed by an overview of interactions between these responses observed in systemic AID. We discuss (i) the effects of GM-CSF-secreting Th17.1 cells and type 1 interferons on CCR2^+^ monocytes; (ii) convergence of IL-17 and type 1 interferon signaling on stimulating B-cell activating factor production and the central role of neutrophils in this process; and (iii) synergy between IL-17 and type 1 interferons in the generation and function of tertiary lymphoid structures and the associated follicular helper T-cell responses. Evaluation of these autoimmune-related pathways in TB pathogenesis provides a new perspective on recent developments in TB research.

## 1 Introduction

Tuberculosis (TB) has been responsible for an estimated one billion deaths worldwide over the last 200 years (1), which is more than any other infectious disease caused by a single pathogen. Given its global magnitude, it has been hypothesized that TB particularly contributed to the genetic selective pressure that predisposes for development of autoimmune diseases (AID) (2). This is supported by polymorphism studies of the *TNF* gene, which show an opposite association between susceptibility to TB vs. susceptibility to several AID (3). Additionally, a gender-dependent predisposition to either TB or AID exists with a male predominance among TB patients (4) opposed to increased AID incidences in women (5). The general concept of an inverse relation between infectious diseases and AID is best described by the hygiene hypothesis, which states that diminished exposure to infectious pathogens during childhood increases the chances of developing AID and allergies (6, 7). Also, epidemiologically, the decline in burden of infectious diseases over the last century in industrialized countries is accompanied by increasing rates of AID (8).

Despite support for an inverse relation, similarities between TB and AID have also been identified. TB is even hypothesized to be an infection-induced AID based on the observation that diverse clinical autoimmune phenomena frequently occur in TB patients (9, 10). Furthermore, up to 32% of patients with active TB have elevated autoantibody titers (11, 12). Rational explanations for these findings could be that either TB or AID activate common immunological pathways (10), or protective immunity in TB increases the chance to develop AID (2). In both scenarios, key findings in AID immunology could potentially contribute to our understanding of TB pathogenesis.

**Figure 1:**
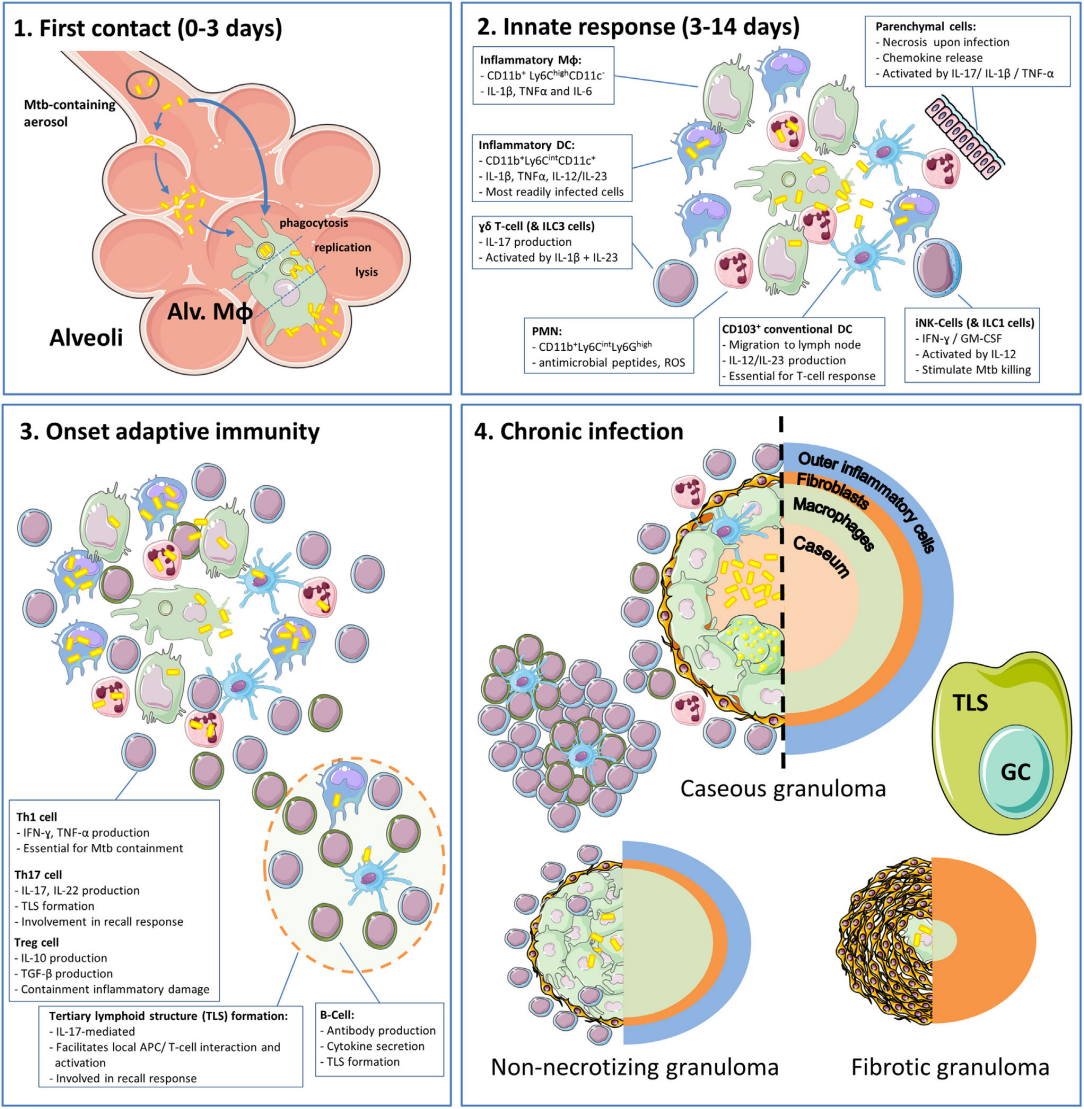
**The phases and cell types involved in the immune response to tuberculosis (TB) in the lungs**. (1) Inhaled Mtb-containing aerosols are deposited deep into the lung, reaching the alveoli (22). Within the alveoli, Mtb are phagocytosed by alveolar macrophages (Alv. MΦ) or infect alveolar epithelial cells prior to ending up in alveolar macrophages (23). Within Alv. MΦ, the bacteria are able to inhibit phagosome–lysosome fusion and replicate until cell lysis ensues, which takes approximately 3–5 days (24). (2) After the initial contact, Mtb encounters infiltrating myeloid cells of which inflammatory dendritic cells and PMN are most readily infected (13, 25). During these early phases, invariate natural killer (iNK) cells and type 1 innate lymphoid cells (ILC1) produce IFN-γ in response to IL-12 and stimulate myeloid cells to kill phagocytosed Mtb. In addition, γδ T-cells and ILC3 produce IL-17. There is increasing appreciation for the role of tertiary lymphoid structures (TLS) and their associated germinal centers (GC) that arise under influence of IL-17 and facilitate optimal activation of myeloid cells and efficient recall responses. During this process, loosely aggregated “innate granulomas” are already formed (26). It should be noted that the roles of ILC1s and ILC3s are based on their general function, which has not yet been formally demonstrated in TB (27). (3) Onset of adaptive immunity in Mtb infection is delayed to *circa* 14 days in mice and up to 6 weeks in humans (13, 22). At this point, distinct T-cell subsets and B-cells migrate to the site of infection and execute their different effector functions. (4) After onset of adaptive immunity, 90–97% of infected individuals develop sustained infection without clinical symptoms termed “latent TB infection” (LTBI) (13). LTBI was initially considered a static phase, but it is now known that this stage is hallmarked by the presence of granulomas in various stages (caseous, non-caseous, and fibrotic) and an ongoing balance between antimycobacterial activity and regulatory mechanisms to minimize immunopathology (13, 28). Cell phenotypes are as present in mouse TB models.

The current paradigm of the host response to Mtb infection is summarized in Figure 1. The indispensable role of IL-12/IFN-γ-mediated Th1 immunity against Mtb has long been recognized (13). However, stimulating Th1 immunity in TB can also result in excessive inflammation (see Box 1). More recently, the contributions of additional immune pathways have been explored, especially the role of type I interferons (T1-IFNs), Th17 immunity (14, 15), and unconventional T cell immunity (16–18). Little is known about the potential interaction between T1-IFNs and Th17 responses in TB, but interesting observations in this regard have been reported for multiple AID (19–21). To determine if these findings are relevant for the understanding of TB pathogenesis, we first review the separate involvements of T1-IFNs and Th17 responses in TB pathogenesis in Sections 2 and 3, respectively. Next, their known interactions in AID are discussed in Section 4. Finally, in Section 5, the potential relevance of these interacting pathways in TB is assessed and integrated into the current understanding of TB pathogenesis.

Box 1 **The dual faces of IFN-γ in tuberculosis (TB) immunity.**

In the current paradigm of a successful host response, lung DCs migrate to the draining lymph node after Mtb contact and induce a robust IL-12-mediated Th1 response (13). This results in migration of IFN-γ-producing CD4^+^ T-cells to the site of infection. Subsequently, activation of macrophages by IFN-γ results in killing of intracellular Mtb, while activated CD8^+^ T-cells lyse infected host cells. Conversely, unsuccessful clearance of infection is due to poor activation of adaptive immunity. This can result from insufficient antigen presentation (29), or from the action of regulatory factors that interfere with Th1 responses such as IL-10 or PDL1-PD1 interaction (13). Paradoxically, the current vaccine bacillus Calmette–Guérin (BCG) induces a strong Th1 response but is only partially effective in protecting against TB (30). Boosting the Th1-inducing potential of BCG by using a modified Ankara virus also has yielded disappointing results (31, 32). Thus, solely stimulating Th1 immunity might not be the solution in TB prevention. This is confirmed in a mouse TB study showing that increasing IFN-γ production by T-cells in the lungs is detrimental to the host due to hyper-inflammation that requires PD-1-mediated suppression to limit pathology (33). In line with this, Mtb-infected mice deficient in PD-1, or mice in which PD-1 is selectively inhibited, display excessive inflammation and disease progression (34, 35). Finally, *ex vivo* studies in human monocyte-derived macrophages show that protective effects of IFN-γ are dependent on multiple factors including time of contact, concentration, and the magnitude of the ensuing microbial challenge (36). Based on these observations, it can be concluded that boosting IFN-γ production and Th1 immunity in TB, besides potentially enhancing protection, can also result in unbalanced inflammation in the lungs that is more harmful to the host than to the pathogen. This emphasizes the need for involvement of additional immunological pathways for optimal protection.

## 2 T1-IFNs in TB

Type I interferons comprise a family of 13 IFN-α subtypes, IFN-β, IFN-ε, IFN-κ, and IFN-ω, which have the shared ability to bind to the IFN-α/β receptor (IFNAR) (37). Other interferons include the single type II interferon, interferon-γ, and the type III interferon family covering three IFN-λ types.

All nucleated cell types are capable of both producing T1-IFNs and responding to them, while type II/III interferons are mostly produced by leukocytes (37). The main function of T1-IFNs is to “interfere” with intracellular infections. Therefore, T1-IFN expression is primarily induced through cytoplasmic pattern recognition receptors (PRRs) and endosomal toll-like receptors (TLRs), which activate distinct interferon regulatory factors (IRFs) that act as transcription factors enabling expression of interferon-responsive genes (38). In contrast, extracellular pathogens trigger surface-bound TLRs that preferentially induce IL-1β and TNF-α through activation of NF-κB.

The role of T1-IFNs in infectious diseases is complex (15, 39–41). T1-IFNs boost the immune system upon pathogen encounter by activating dendritic cells and NK cells and by stimulating both B-cell responses and CD4^+^/CD8^+^ T-cell responses. However, T1-IFNs can also induce anti-inflammatory responses to control immune-mediated tissue damage during chronic infections. These contradictory effects of T1-IFNs in different situations can likely be ascribed to the heterogeneity of the T1-IFNs family, downstream activation of different STAT homo/heterodimers after binding to IFNAR (38, 42) and to differential priming of cells prior to induction of T1-IFN signaling (43).

### 2.1 T1-IFNs in Human TB

When recombinant or purified T1-IFNs became available as therapeutic agents in the 1980s, different applications have been established based on their antiviral, immune-stimulating, and suppressive effects. These include treatment of viral infections (e.g., IFN-α treatment of hepatitis B/C infections), AID [e.g., IFN-β treatment for multiple sclerosis (MS)], and various malignancies (44). Based on their well-described immune-stimulating effect, the use of T1-IFNs as adjuvant to antibiotic treatment for patients with active TB has also been explored (see Table 1). All studies found a positive influence of adjuvant T1-IFN therapy on clinical outcomes in active TB (45–49). Conversely, IFN-α treatment without concomitant antibiotic treatment, e.g., for hepatitis C, has been described to cause reactivation of latent TB (50–57). While reactivation of latent TB and treatment of active TB are two distinct clinical situations, the latter finding suggests an unfavorable role for T1-IFNs in TB pathogenesis.

**Table 1:** **Effect of type I interferons supplementation in human tuberculosis (TB)**.

Study design	Regimen	Outcome	Side effects	Reference
Open parallel, susceptible Mtb strain, HIV (−), *N* = 20 (2 × 10), 2 months treated	HRZE vs. HRZE + IFN-α	– Less fever on days 3 and 4 after start treatment in HRZE + IFN-α group	No adverse effects reported	Giosue et al. (45)
		– Increases in total lymphocytes and HLA DR1^+^ cells after 2 months only in HRZE + IFN-α group		
		– Reduction in HRCT score only in HRZE + IFN-α group		
		– Stronger reduction of pro-inflammatory cytokines in BALF after 2 months treatment in HRZE + IFN-α group		
Patients treated prior for 3–12 years, MDR strain, HIV (+), *N* = 5, 12 weeks treated	Anti-TB treatment + IFN-α	– 2/5 complete response	Flu-like symptoms in 4/5 patients, not needing treatment	Palmero et al. (46)
		– 1/5 partial response		
		– 2/5 no response		
		– Increase of NK (% cytotoxicity) in all patients after 12 weeks		
Patients treated prior for 6 months, MDR strain, HIV (−), *N* = 7, 9 weeks treated	DOT + IFN-α	– Significant drop (*p* = 0.02) in Mtb loads at the end of a 9-week IFN-α treatment course	No adverse effects reported	Giosue et al. (47)
		– Significant increase (*p* = 0.03) in Mtb loads after stop of IFN-α treatment		
		– Significant drop in IL-1β, IL-6, TNF-α, and IFN-γ pro-inflammatory cytokines; IL-4 and IL-10 showed inconsistent changes		
Parallel, patients treated prior for 6 months with DOT, MDR strain, HIV (−), *N* = 12 (2 × 6), 8 weeks treated	1. DOT 2. DOT + IFN-α	– After 8 weeks, all five subjects of the case group became sputum smear negative; the control group remained smear positive (*p* = 0.012)	4 subjects mild arthralgia and myalgia, flu-like symptoms in all subjects	Mansoori et al. (48)
		– Evaluation of smear results after 6 months showed two smear-negative subjects in the case group while all controls were smear positive (*p* = 0.132)		
Case report, MDR strain, HIV (−), *N* = 1, 2 months treated	HRZE + IFN-α	– Two months after initiation of therapy, sputum smears became negative, the patient’s clinical and radiological findings strikingly improved. During 4-year follow-up, all consecutive sputum cultures remained negative	No adverse effects reported	Zarogoulidis et al. (49)

*Green text indicates a host-beneficial effect in TB; BALF, bronchoalveolar lavage fluid; DOT, directly observed therapy (antibiotic TB treatment); HRCT, high-resolution computed tomography; HRZE, isoniazid, rifampicin, pyrazinamide, and ethambutol; MDR, multidrug resistant*.

In 2010, an interferon-inducible transcriptional signature was reported in circulating leukocytes of TB patients, thus linking increased T1-IFN signaling with active disease (58). This finding has been validated in several independent studies (59–62). A meta-analysis confirmed statistical significance but found a less dominant role for T1-IFN-related genes than expected (63). This is ascribed to the involvement of signaling components downstream of the T1-IFNs receptor in multiple overlapping intracellular pathways. Also, association studies do not necessarily implicate a causally detrimental effect of T1-IFNs in TB pathogenesis. In line with this, T1-IFN responses show potential as biomarkers or diagnostic tool for risk of active disease, but their functional involvement during TB progression in patients is not yet understood (62).

### 2.2 Preclinical Studies in Mice Support a Detrimental Role of T1-IFNs during Acute TB

A causal relationship between T1-IFN signaling and TB disease severity was first suggested in 2001 when IFN-α levels in the lungs of Mtb-infected mice were shown to be associated with Mtb strain virulence (64). Several approaches have been used to verify this relationship between increased T1-IFN signaling and unfavorable disease outcome. Blocking the T1-IFN signaling pathway through use of IFN-α/β receptor knockout (IFNAR^−/−^) improves survival, but only when applied on the background of mouse strains in which acute TB is lethal, such as the A129 strain (65). In IFNAR^−/−^ mice with a relatively TB-resistant C57BL/6 background, survival rates were similar to wild-type mice, but mycobacterial loads in the lungs were lower (66–69). One study actually observed increased loads in the lungs (70) (Table 2).

**Table 2:** **Interference with T1-IFN signaling in preclinical tuberculosis (TB) studies**.

Mouse back ground	Intervention	Mtb strain	Survival	Mtb load	Reference
A129	IFN-α/β receptor knockout (IFNAR^−/−^)	HN878, W4, CDC1551, 100–200 CFU, aerosol	Better survival against CDC1551	No data	Manca et al. (65)
			Trend toward better survival against HN878		
B6D2/F1	Anti-IFN-α/β antibody	HN878, 100–200 CFU, aerosol	Better survival against HN878	No differences up to day 100	Manca et al. (65)
B6/129	IFNAR^−/−^	H37Rv, HN878, CSU 93, CSU 123 50–100 CFU, aerosol	No differences in survival after infection with all strains	Lower Mtb loads in lungs after infection with all strains up to day 150	Ordway et al. (66)
B6	IFNAR^−/−^	Erdman, 10^6^ CFU, i.v. injection	No data	No differences in lung until day 20	Stanley et al. (67)
				Lower Mtb loads in spleen at day 10 and day 20	
B6	IFNAR^−/−^	H37Rv, 100 CFU, aerosol	No differences up to day 70	Lower Mtb loads in lungs at day 18,	Desvignes et al. (68)
				no differences at day 25	
129S2	IFNAR^−/−^	H37Rv, 200 CFU, aerosol	Improved survival	Lower Mtb loads at day 21	Dorhoi et al. (69)
B6	IFNAR^−/−^	H37Rv, 500 CFU, aerosol	No differences in survival up to day 90	Lower Mtb loads at day 21	Dorhoi et al. (69)
B6.SJL	IFNAR^−/−^	H37Rv, 100–150 CFU, aerosol	No differences in survival up to day 90	No data	Mayer-Barber et al. (71)
B6/129	IFNAR^−/−^	Erdman, 100 CFU, aerosol	No data	Higher Mtb loads in lungs on day 10, day 20, and day 40	Cooper et al. (70)
				Equal loads at day 80	

*Green text indicates a host-beneficial effect in TB, while red indicates harmful effects*.

*i.v., intravenous; CFU, Colony Forming Units*.

In a second approach, Mtb-infected mice were supplemented with T1-IFNs after start of infection or treated with the TLR3-ligand poly-ICLC, which stimulates T1-IFN production and signaling (64, 72). Both studies showed increased mortality and higher mycobacterial loads in the supplemented groups, which were not observed when T1-IFNs or poly-ICLC were administered to Mtb-infected IFNAR^−/−^ mice. Finally, in a third approach, mice were primed with a T1-IFN-inducing influenza virus prior to TB infection, which led to enhanced mycobacterial growth and reduced survival (73).

Enigmatically, reduced mycobacterial loads in IFNAR^−/−^ mice are primarily observed in the acute phase of infection in which T1-IFNs are considered immune stimulating. No differences in survival or long-term control of infection were found in C57BL/6 IFNAR^−/−^ mice compared to wild type. In support of this notion, T-cell analyses in several of the abovementioned studies convincingly excluded an effect of increased or decreased T1-IFN signaling on the adaptive immune response (68, 69, 72). Notably, none of these studies addressed the effect of T1-IFNs as adjunct treatment to antibiotics, which was shown to be beneficial in TB patients (Table 1).

### 2.3 Mtb Actively Induces T1-IFNs

Multiple studies indicate that Mtb employs both active and passive mechanisms to induce T1-IFNs (74–76). The mycobacterial ESAT-6 secretion system (ESX-1) and its 6 kDa early secretory antigenic target (ESAT-6) are essential in this process, as mycobacteria lacking ESX-1 fail to induce T1-IFN production (67, 77–81). ESAT-6 can disrupt the phagosomal membrane, which allows translocation of mycobacteria and mycobacterial products from the phagosome into the cytosol (78, 82).

Mycobacteria actively secrete several T1-IFN-inducing compounds, including double-stranded (ds)DNA and the bacterial second messenger cyclic-di-AMP (83). These compounds are recognized by different cytosolic PRRs, including cGAS (80), IFI-204 (78), AIM2 (84), and possibly NOD2 (77), although data on the latter are conflicting (67, 78). Activation of these cytosolic PRRs converges to activate “STimulator of INterferon Genes” (STING), which subsequently forms a complex with TANK-binding kinase 1 (79). This STING–TBK1 complex activates IRF3, leading to IFN-β production in mice (81) as well as human dendritic cells (74). IRF3^−/−^ mice are poor producers of IFN-β and more resistant to Mtb infection, which supports a negative role for T1-IFNs in TB pathogenesis (78).

However, the overall picture is more complex. IRF3^−/−^ mice are more resistant to Mtb infection, but mice deficient in the cytosolic PRR cGAS, upstream of IRF3, show diminished control of chronic Mtb infection (79). This can be traced back to a concomitant reduction in autophagy, which is also dependent on the cGAS-induced activation of the STING–TBK1 axis, but independent of IRF3. In line with this, mice infected with an Mtb strain that induces higher amounts of cyclic-di-AMP, thus stimulating both IRF3-mediated IFN-β production and STING–TBK1-mediated autophagy, show improved survival despite increased IFN-β levels (83). Taken together, this suggests that pro-mycobacterial effects of stimulating the cytosolic PRR/STING/IRF3/IFN-β axis by mycobacteria might be outweighed by the antimycobacterial effects of the PRR/STING/autophagy pathway.

Autocrine or paracrine IFN-β-signaling induces IRF7 and leads to the production of IFN-α in human dendritic cells (74). In line with this, injection of recombinant IFN-β in mice induces IFN-α production (85). Alternatively, myeloid cells and particularly plasmacytoid dendritic (pDC) cells are capable of directly activating IRF7-mediated IFN-α production after recognition of Mtb, particularly by endosomal TLR9 (86). In TB, this TLR9-IRF7 pathway is studied to lesser extent than the cytosolic PRR–IRF3 axis (87). This is possibly due to the dependence of T1-IFN-mediated pathogenic effects in mice on ESX-1, which induces IRF3 rather than IRF7 as explained above (67). However, IRF7 is recognized as commonly induced transcription factor by multiple clinical Mtb strains in alveolar epithelial cells (88). Moreover, TLR9^−/−^ mice succumb earlier to high-dose Mtb infection than wild-type mice, which suggests a role for the TLR9/IRF7/IFN-α axis in TB as well (89).

### 2.4 T1-IFNs Drive the Influx of Mtb-Permissive Myeloid Cells during Acute Infection

Most studies in mouse TB models found significant functional effects of T1-IFNs specifically on CD11b^+^Gr1^int^ myeloid cell populations (68, 69, 72). This population comprises monocyte-derived Ly6C^high^CD11c^−^CCR2^high^ inflammatory macrophages (iM) and Ly6C^int^CD11c^+^CCR2^int^ inflammatory dendritic cells (iDC), but not CD11b^+^Gr1^high^ PMN (90). This is an important distinction, as T1-IFNs actively inhibit PMN influx, as discussed in more detail in Section 2.4.3.

Inflammatory macrophages and iDC have been identified as major contributors to disease progression in mouse TB models (91–93). Several lines of evidence suggest that T1-IFNs regulate the influx of these cells and play a role in their functional impairment to resist Mtb. This interference with protective immunity is multifaceted and concerns four important interactions, which will be reviewed separately: (1) T1-IFNs mediate the influx of iM and iDC. (2) T1-IFNs inhibit IL-1β responses by these cells, which are essential in the initial host responses to Mtb. (3) Prolonged IL-1β signaling can also cause excessive inflammation and thus requires regulation during later phases. This can be mediated by T1-IFNs but also by IFN-γ through functionally different routes. (4) T1-IFNs and IFN-γ show a complex interplay in the activation of iM and iDC.

#### 2.4.1 T1-IFNs Mediate the Influx of iM and iDC

Mtb-infected mice treated with the T1-IFN-inducing compound poly-ICLC show increased numbers of iM and iDC in the lungs, which are 10 times more permissive to Mtb infection than their counterparts in PBS-treated mice (72). Others confirmed that signaling through IFNAR indeed augments the recruitment of Mtb-permissive iM and iDC into the lungs (69). Mechanistically, IFNAR-dependent expression of the chemokine CCL2 mediates the influx of CCR2^+^ monocytes that differentiate into iM and iDC (72). Both myeloid and parenchymal cells can produce CCL2 in response to T1-IFNs, but parenchymal cells appear the main source of this chemokine (94–96). Expression of CCL2 is reduced in the lungs of IFNAR^−/−^ mice, and the pathogenic effects of poly-ICLC treatment are absent in Mtb-infected CCR2^−/−^ mice (72). Thus, preclinical TB studies indicate that T1-IFNs stimulate the influx of CCR2^+^ monocytes, but not PMN, to the site of infection in a CCR2-dependent way *via* the induction of CCL2 in parenchymal cells (74–76).

#### 2.4.2 T1-IFNs Inhibit IL-1β Responses during Acute TB

Type I interferons not only stimulate the influx of CCR2^+^ monocytes but also stimulates their differentiation into Mtb-permissive iM and iDC (72, 75, 76). This can be traced back to a cross talk between T1-IFNs and IL-1β (71, 90). iM and iDC are the major sources of IL-1β in the lungs Mtb-infected mice, and IL-1β plays a crucial role in the acute host response to Mtb infection (71, 90). IL-1β augments TNF-α-stimulated Mtb killing and increases prostaglandin E_2_ (PGE_2_) production by upregulating cyclooxygenase-2 (COX2/PTGS2) (71, 97, 98). PGE_2_ is involved in control of intracellular Mtb replication but also prevents necrotic host cell death (99). In accordance, *Ptgs2^−/−^* mice, unable to produce PGE_2_, are more susceptible to Mtb infection than wild type mice, but to a lesser degree than IL1^−/−^ mice. Further, information on PGE_2_ in TB is given in Box 2.

Box 2 **The dual faces of prostaglandin E_2_ (PGE_2_) in tuberculosis (TB) immunity.**

Prostaglandin E_2_ is generally considered a pro-inflammatory mediator and indispensable for the induction of fever, which is a hallmark symptom of active TB (100, 101). The anti-inflammatory effects of prostaglandin synthase (COX-) inhibitors such as NSAIDs underline this notion. However, high levels of PGE_2_ can also exert immunosuppressive effects as they stimulate alternative activation of macrophages (102), inhibit bactericidal activity (103), and promote production of IL-10 (104). Moreover, high PGE_2_ levels can stimulate the development of myeloid-derived suppressor cells with inhibitory effects on adaptive immune cells (104, 105). Finally, PGE_2_ inhibits IL-12 production by DCs and IFN-γ production by T-cells, thereby promoting Th2/Th17 immunity (106, 107).

In the serum and bronchoalveolar lavage fluid of TB patients, PGE_2_ levels were found to be elevated (71, 108, 109), and polymorphisms in the PGE_2_ receptor EP2 are associated with TB-susceptibility (110). Experimentally, one mouse study showed that low PGE_2_ levels in the acute phase of infection are essential for iNOS-mediated control of Mtb (111). Also, PGE_2_ plays an important role during acute TB since the PGE_2_-producing enzyme COX2 competes for arachidonic acid substrate with 5-lipoxygenase, which produces leukotrienes and lipoxins. Hereby, PGE_2_ prevents necrotic cell death thus benefiting the host (71). Opposed to the protective role of low PGE_2_ levels during acute disease, PGE_2_ levels are higher during the chronic phase of TB, and these concentrations contribute to disease by suppressing IFN-γ, TNF-α, and iNOS (111). Notably, the cellular source of PGE_2_ appears to differ between acute and chronic TB. During the acute phase of infection, inflammatory myeloid cells are the main source of PGE_2_, while foamy macrophages are strong producers of PGE_2_ during the chronic phase of disease (112). In line with a detrimental effect of high PGE_2_ levels in the chronic phase, foamy macrophages are typically associated with disease progression (113).

**Figure 2:**
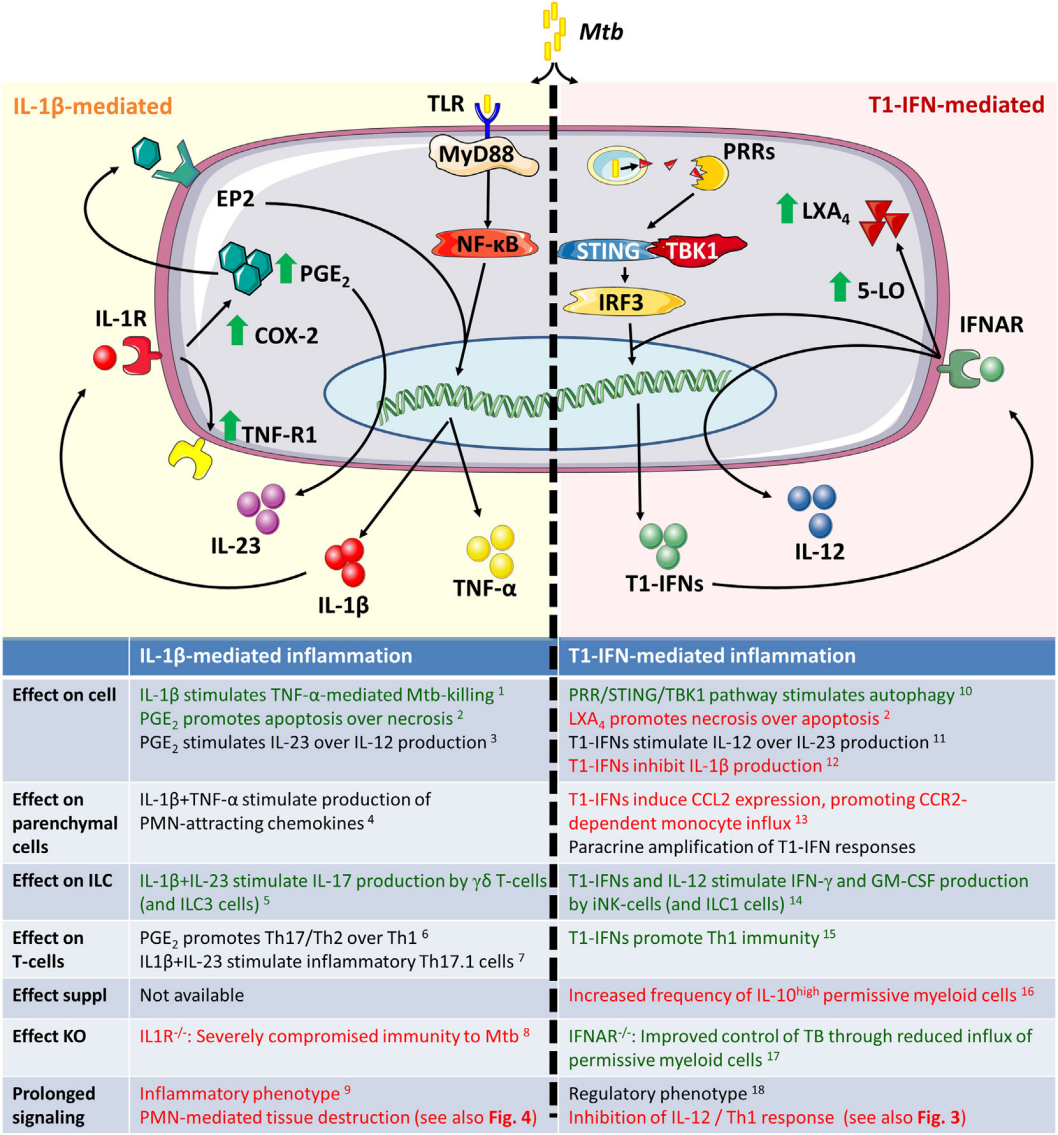
**Inflammatory responses during acute infection in naïve inflammatory macrophages and dendritic cells**. Green text indicates a beneficial host effect during Mtb infection and red indicates a detrimental effect. Mtb, *Mycobacterium tuberculosis*; PRR, pattern recognition receptor; STING, STimulator of INterferon Genes; TBK1, tank-binding kinase 1; IRF3, interferon regulatory factor 3; 5-LO, 5-lipoxygenase; COX-2, cyclooxygenase 2; PGE_2_, prostaglandin E_2_; EP2, prostaglandin E_2_ receptor 2; ILC3, innate lymphoid cells type 3. ^1^Jayaraman et al. (97), ^2^Chen et al. (116), ^3^Shi et al. (107), ^4^Di Paolo et al. (98), ^5^Lockhart et al. (117), ^6^Boniface et al. (106), ^7^El-Behi et al. (118), ^8^Fremond et al. (119), ^9^Mishra et al. (120), ^10^Watson et al. (81), ^11^Fleetwood et al. (94), ^12^Mayer-Barber et al. (71), ^13^Antonelli et al. (72), ^14^Une et al. (121), ^15^Longhi et al. (122), ^16^Manca et al. (64), ^17^Manca et al. (65), and ^18^Mayer-Barber et al. (90).

Type I interferons inhibit the expression and production of IL-1β and simultaneously increase the expression of 5-lipoxygenase (5-LO), which is a competitive enzyme for COX2 in the arachidonic acid metabolism (71, 90, 114, 115). As a result, IFNAR signaling causes a shift from COX2-mediated PGE_2_ production to an increase in the 5-LO products such as lipoxin A_4_ (LXA_4_) and leukotriene B_4_ (LTB_4_), which render cells more susceptible to necrotic cell death (71, 116). Pharmacological intervention in this process by administrating the 5-LO inhibitor Zileuton to Mtb-infected mice, improved disease outcomes during acute infection to similar extent as observed in IFNAR^−/−^ mice (71). An overview on the balance between IL-1β and T1-IFNs is given in Figure 2.

#### 2.4.3 Prolonged IL-1β Signaling Causes PMN-Mediated Tissue Damage and Is Regulated by Both T1-IFNs and IFN-γ

The cross talk between T1-IFNs and IL-1β influences disease outcome in TB (71). However, this does not fully explain the harmful effects of T1-IFNs observed in TB. Most importantly, although IL-1β production is essential for protective immunity in the acute phase of disease in TB, it requires strict regulation as unchecked IL-1β signaling in TB can result in excessive PMN-mediated tissue damage (120, 123). Also, as explained in Box 2, IL-1β-mediated PGE_2_ production is protective during acute disease but appears to have a detrimental effect during chronic disease. Finally, inflammatory mediators associated with continuing infection, e.g., GM-CSF, predispose for IL-1β production over T1-IFNs by iM and iDC (36, 94, 124–126). This reflects an increasing need over time to limit IL-1β-mediated inflammatory responses.

To prevent PMN-mediated inflammation caused by excessive IL-1β signaling, the expression and production of IL-1β is inhibited not only by T1-IFNs but also by IFN-γ (90, 120). In line with this, both T1-IFNs and IFN-γ can inhibit PMN influx (127–131). T1-IFNs and IFN-γ can both reduce pro-IL-1β gene expression and increase the expression of soluble antagonists for the IL-1 receptor (114, 132).

Despite the abovementioned functional similarities between T1-IFNs and IFN-γ in IL-1β inhibition, mechanistic differences exist between these IFN types in mediating this effect.

*Ex vivo* studies in human iM and iDC demonstrate that IFN-β inhibits IL-1β production more potently than IFN-γ (90, 114). One explanation might be that IFN-γ inhibits IL-10, while T1-IFNs induce IL-10, which contributes to the inhibition of IL-1β production (90, 114, 115). Additionally, an IL-10-independent inhibition of IL-1β by T1-IFNs was recently identified (129). T1-IFNs induce cholesterol 25-hydroxylase, which potently reduces IL-1β transcription and broadly represses IL-1-activating inflammasomes. In contrast, IFN-γ inhibits IL-1β by increasing intracellular nitric oxide in an iNOS-dependent way (120). This prevents NLRP3 inflammasome activation and cleavage of pro-IL-1β into IL-1β. In contrast to the mechanisms exerted by T1-IFNs, IFN-γ-induced iNOS not only limits IL-1β-mediated inflammation but also markedly enhances the bactericidal potential of iM (120). Conversely, T1-IFNs suppress iNOS production (90). Based on the stimulation of iNOS by IFN-γ and the inhibition of iNOS by T1-IFNs, it appears that iDC are more sensitive to T1-IFN signaling and iM to IFN-γ when both types of interferon are present. T1-IFN-mediated inhibition of iNOS appears to occur primarily in iDC, since iDC only expressed iNOS in IFNAR^−/−^ mice during viral infection, while iM appear more sensitive to IFN-γ and are the main source of iNOS in wild-type mice (131).

When taken together, these data suggest that IL-1β inhibition by either T1-IFNs or IFN-γ has strong implications on the bactericidal potential of iM and iDC. Furthermore, T1-IFNs interfere with the induction of iNOS by IFN-γ, particularly in iDC. This fits the observation that IFN-γ only inhibits IL-1β production by iM but not iDC in mouse TB models (90). Notably, iDC are the most readily infected cells in the lungs of Mtb-infected mice (25) and are present in larger numbers than iM during Mtb infection (72, 133).

#### 2.4.4 The Interplay between T1-IFNs and IFN-γ

During direct contact with Mtb through TLRs, endogenous T1-IFN signaling through IRF3 promotes IL-12 production by iDC over IL-23 (see also Figure 2; Box 3) (94, 134, 135). This early IL-12 signaling is required to induce IFN-γ production by innate lymphoid cells (ILC) such as NK cells and possibly ILC1s (136, 137). However, exogeneous T1-IFNs or T1-IFN signaling in the absence of TLR stimulation can also inhibit IL-12 production by iDC (115, 138). This inhibition of IL-12 by T1-IFNs occurs particularly through induction of IL-10 (15). T1-IFNs also inhibit the responsiveness of iDC to IFN-γ-mediated activation, which is required for Mtb killing. This occurs partially by reducing the expression level of IFN-γ-receptor on the cell surface, but primarily through induction of an IL-10^high^ regulatory phenotype in which antimicrobial pathways by IFN-γ are not readily activated, as discussed below (90, 115, 131, 139–141).

Box 3 **IL-12 or IL-23 production by dendritic cells?**

IL-12 and IL-23 are heterodimeric cytokines composed of a common p40 subunit, coupled with either a p35 subunit in IL-12 or a p19 subunit in IL-23. Both IL-12 and IL-23 are produced in particular by stimulated dendritic cells and to lesser degree by macrophages. The preferential production of IL-12 or IL-23 by these cells is multifactorial. Increased levels of PGE_2_ support IL-23 production over IL-12 (106, 107, 142). Activation of TLR2 and TLR4 also stimulates IL-23 production over IL-12, especially when NOD2 is simultaneously activated (143, 144). On the other hand, TLR9 and TLR3 agonists preferentially induce IL-12 (135, 145, 146). Downstream of PRRs, activation of IRF 4 and 5 favor induction of IL-23, while IRF 1, 3, and 7 induce IL-12 (135, 147). In line with this, T1-IFN-mediated IRF3 activation and IFN-γ-mediated IRF-1-activation both favor IL-12 production (148, 149).

IL-4 also favors IL-12 production and inhibits IL-23 production, especially in combination with IFN-γ or GM-CSF (150, 151). Finally, an important pathway that promotes IL-12 over IL-23 is ligation of the co-stimulatory molecule CD40 by CD40L on activated T-cells or by agonist antibodies (152). Taken together, IL-23 is induced in the presence of pathogens and innate signaling in the acute phase of infection, while onset of adaptive immunity with increased levels of IFN-γ and/or IL-4 shifts the balance toward IL-12 (153).

Recent findings might explain the mechanism behind this paradox where T1-IFNs initially support IL-12-mediated IFN-γ production by NK cells but can also induce an IL-10^high^ phenotype in iDC, which interferes with IL-12 production and prevents IFN-γ-mediated activation. It has been observed in different mouse models, including Mtb-infected mice, that T1-IFNs can only induce an IL-10^high^ regulatory phenotype in monocyte-derived DCs (iregDC) if these cells have been primed previously by IFN-γ (43). IFN-γ-primed DCs that did not receive T1-IFN signaling differentiated into iDC that stimulated robust T-cell responses. This phenomenon of monocyte priming by IFN-γ has been demonstrated to occur in the bone marrow (154). During gut infections, local production of IL-12 in mucosa-associated lymphoid tissue stimulates bone-marrow-resident NK cells to produce IFN-γ as early as 3 days post infection (154). This results in a uniform presence of an IFN-γ-primed signature of Ly6C^high^ monocytes in the circulation at day 6. Furthermore, IFN-γ indeed primed these monocytes toward a regulatory phenotype, as they more effectively produced IL-10 in response to bacterial ligands (154). We speculate that a similar mechanism of IFN-γ priming is likely to be involved in pulmonary infections.

**Figure 3:**
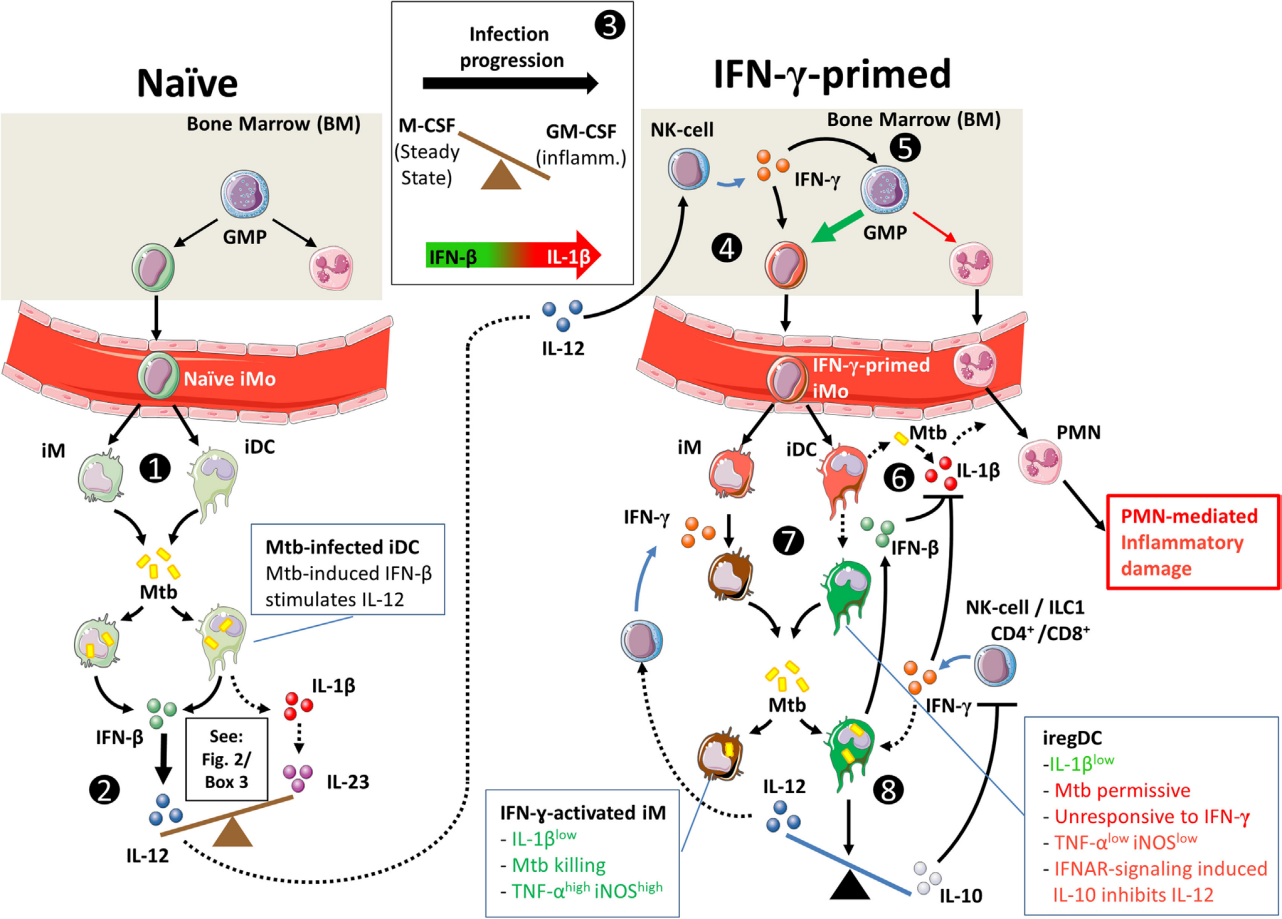
**Hypothetical interplay between type I interferons (T1-IFNs) and IFN-γ in monocyte priming and shaping of the immune response in Mtb infection**. Dashed lines indicate speculations in the context of pulmonary Mtb infection; solid lines indicate shown pathways in human and/or in animal models. (1) T1-IFN induces migration of CCR2^+^ monocytes (iMo) from the bone marrow to the lungs of Mtb-infected mice under influence of CCL2 (72). Locally, these cells develop into CD11b^+^Ly6C^high^ inflammatory macrophages (iM) and CD11b^+^Ly6C^int^ inflammatory dendritic cells (iDC) (43). (2) As shown in Figure 1, naïve iM and iDC can initiate either IL-1β-mediated inflammation or T1-IFN-mediated inflammation. Mtb actively triggers intracellular pattern recognition receptors to induce a T1-IFN-mediated response. (3) Additionally, iM and iDC in the naïve situation have differentiated under influence of M-CSF, which makes them more responsive to T1-IFN signaling (94). During progression of Mtb infection, GM-CSF levels rise and increase the potential for IL-1β production by iM and iDC (94, 125, 155, 156). (4) Similar to the situation in gut infection, we propose that in tuberculosis (TB) IL-12 production in the lungs stimulates IFN-γ production by bone-marrow-resident NK cells, which locally primes monocytes (154). IFN-γ priming of monocyte-derived iDC is necessary for T1-IFNs to induce a regulatory (iregDC) phenotype in iDC in the lungs (43). (5) Additionally, IFN-γ stimulates monopoiesis over granulopoiesis by granulocyte/macrophage progenitor cells (128). (6) As Mtb infection progresses and GM-CSF levels increase, iM and iDC readily produce IL-1β [see also (3)] (124, 155), which can lead to PMN-mediated inflammatory damage in TB (120). (7) IL-1β production can be inhibited in response to either IFN-γ or IFN-β through mechanistically distinct pathways that differently affect Mtb killing. (8) Signaling through IFN-α/β receptor in IFN-γ-primed iDCs induces IL-10 production (43, 115, 131), inhibits IL-12 production (115), and makes these cells unresponsive to activation by IFN-γ (43, 115, 141), which together interfere with protective immunity during acute Mtb infection.

These data suggest interplay between T1-IFNs and IFN-γ as proposed in Figure 3. T1-IFNs initially induce IFN-γ responses by promoting IL-12 production in naïve cells as shown in Figure 2. These IL-12 responses stimulate IFN-γ production by ILC not only locally but also systemically, which results in IFN-γ priming of monocytes in the bone marrow. Once IFN-γ production is initiated, T1-IFNs mediate a regulatory function by inducing an IL-10^high^ phenotype in IFN-γ-primed iDC. This prevents further production of IL-12 by these cells, inhibits their activation by IFN-γ, and results in an Mtb-permissive phenotype.

### 2.5 Summary: The Role of T1-IFNs in TB

Several modest clinical successes have been shown with IFN-α supplementation adjunct to antibiotic TB treatment (Table 1). However, case reports of TB reactivation under IFN-α treatment without concomitant antibiotics have put T1-IFNs in a negative spotlight (50–57). Furthermore, a T1-IFN transcriptional signature in circulating leukocytes is associated with active TB. Nevertheless, the functional role of T1-IFNs in TB patients remains to be determined (62).

Preclinical studies in mice support a detrimental role for T1-IFN in the acute phase of Mtb infection. T1-IFN signaling was associated with increased mortality in Mtb-susceptible mouse strains and higher Mtb loads in the lungs in most studies (Table 2).

However, it should be noted that most of these preclinical studies do not unequivocally support a harmful effect of T1-IFNs during the chronic phase of disease based on mortality, Mtb loads, or differences in adaptive immunity.

In support of a pathogenic role of T1-IFNs during acute infection, mycobacteria actively induce T1-IFNs by triggering cytosolic PRRs. This leads to IFN-β production in an IRF3-dependent way. Subsequently, T1-IFNs mediate the CCL2/CCR2-dependent migration of iM and iDC into the lungs (72). In these cells, interference of T1-IFNs with IL-1β and PGE_2_ as shown in Figure 2 can lead to an altered metabolism of arachidonic acids that leaves cells more vulnerable to necrotic cell death (71). However, sustained IL-1β signaling itself carries the risk of excessive inflammation in TB and not only T1-IFNs but also IFN-γ inhibits IL-1β to prevent excessive PMN-mediated inflammation (120). T1-IFNs inhibit IL-1β more effectively than IFN-γ but stimulate an IL-10^high^ Mtb-permissive phenotype (72, 90).

Next to their shared ability to inhibit IL-1β, an interesting interplay between T1-IFNs and IFN-γ exists in TB as summarized in Figure 3. Two recent findings that are of particular interest include the observation that T1-IFNs can only induce an IL-10^high^ phenotype in IFN-γ-primed cells (43) and the inductive role of T1-IFNs in early IL-12 signaling, which is required for IFN-γ priming in the bone marrow (154). Further, research into this complex interplay between T1-IFNs and IFN-γ during early host responses in TB would be highly interesting given the T1-IFN-inducing capacities of Mtb and the shaping effect of early T1-IFN or IFN-γ signaling on the ensuing immune response.

## 3 The Th17 Response in TB

As discussed in the previous paragraph, T1-IFNs induce IL-12 production by iDC, while IL-1β induces IL-23. Other factors also influence production of IL-12 or IL-23 (see Box 3). IL-12 is essential for the induction of IFN-γ responses in TB, but IL-1β is protective during acute TB despite inducing IL-23 over IL-12. Similar to the requirement of IL-12 for Th1 responses, IL-23 is essential for establishing Th17 immunity (157–159). Here, we review the effect of IL-23 signaling and the Th17 response in TB.

### 3.1 Introduction to the Th17 Response

The Th17 response is distinct from classical cell-mediated Th1 immunity or B-cell-stimulating Th2 responses and is often associated with a potent inflammatory response and tissue damage (159). Th17 cells display a high degree of plasticity and their ability to express signature markers of other T-helper lineages makes it difficult to establish their exact role in disease. Four different subsets of Th17 cells have been described to date with ranging inflammatory potential (160). On one side of the spectrum are highly inflammatory and often pathogenic IFN-γ/GM-CSF-producing Th17.1 cells that result from prolonged IL-1β and IL-23 signaling (161). On the other side are IL-10-producing Th17 cells, which can even transdifferentiate into regulatory T-cells and contribute to resolution of inflammation (162).

Despite the plasticity in cytokine production, IL-17 remains the hallmark cytokine of the Th17 response. Next to Th17 cells, γδ T-cells and ILC3 can also produce IL-17 in response to IL-23 and IL-1β (27, 117, 163). IL-17 exerts its effects primarily on nearby parenchymal cells and to lesser extent on hematopoietic cells, which is distinct from Th1 and Th2 cytokines like IFN-γ and IL-4 (159). In parenchymal cells, IL-17 primarily stimulates the production of the chemokines that attract PMN (164). However, it should be noted that IL-17 alone is a poor inducer of these chemokines and that synergistic activation by inflammatory ligands such as IL-1β, TNF-α, or GM-CSF markedly increases the effects of IL-17 (164, 165).

### 3.2 The Th17 Response in Human TB Infection

The exact role of the Th17 response in human TB remains a topic of debate (13, 14, 166). Polymorphisms in genes encoding IL-17 are associated with susceptibility to pulmonary TB, which indicates a role for this cytokine in TB (167–170). However, these findings could not be reproduced in different demographic settings (171, 172).

Analyses of Th17 responses in peripheral blood mononuclear cells (PBMC) from TB patients do not show uniform results either. Direct *ex vivo* analyses of unstimulated circulating CD4^+^ T-cells show that active TB (ATB) is associated with reduced frequencies of circulating Th17 cells compared to latent TB infection (LTBI) (173, 174). However, serum IL-17 levels do not differ between ATB and LTBI, and IL-17 is undetectable in the bronchoalveolar lavage fluid during both stages of disease (174, 175).

Different studies report PBMC stimulation assays with Mtb-specific antigens showing either increased or reduced Th17 responses in ATB compared to LTBI (Table 3). These diverse findings are similar to those observed in IFN-γ response assays (IGRA), in which the levels of IFN-γ often also cannot discriminate between ATB and LTBI (176, 177). Interestingly, both Th1 and Th17 cells appear functionally inhibited in ATB patients by a PD-1-mediated immunosuppressive state (178–181). In accord, reductions in PD-1 expression under TB treatment restored both Th1 and Th17 responses (182).

**Table 3:** **IL-17 responses in patients with active TB (ATB) compared to latent tuberculosis infection (LTBI)**.

	Increased in ATB	No difference	Reduced in ATB
% of IL-17^+^CD4^+^ T-cells			
Short incubation (0–48 h)	Basile et al. (183)^a^	Marin et al. (184, 185)	Scriba et al. (175); Perreau et al. (186)
Long incubation (72–144 h)	Jurado et al. (187); Marin et al. (184)	Cowan et al. (174); Marin et al. (185)	Perreau et al. (186); Heidarnezhad et al. (188)
*Ex vivo* IL-17 production	Jurado et al. (187); Xu et al. (189)	Sargentini et al. (190); Cowan et al. (174); Kim et al. (191)	Kumar et al. (192); Nunnari et al. (193); Bandaru et al. (182)

*^a^Only in MDR-TB*.

Taken together, systemic Th17 responses in TB patients demonstrate similar variability as observed for IGRA studies. Both are unable to distinguish ATB from LTBI. How these systemic responses relate to local host responses in the lungs has not been characterized in TB patients.

### 3.3 Preclinical Studies in Mice Support a Protective Role for IL-23 and IL-17 in TB

Based on mortality and mycobacterial loads, studies in Mtb-infected mice support a protective role for IL-23 and IL-17 in TB, but only during later stages of disease (Table 4).

**Table 4:** **Th17-related effects in preclinical tuberculosis (TB) studies in mice**.

Mice (age, weeks)	Intervention	Mtb strain, route	Survival	Mtb load (vs. wild-type mice)	Immunological effect	Reference
B6 (6–12)	IL23 p19^−/−^	H37Rv (100 CFU), aerosol	No data	No difference in lungs	No IL-17-producing cells in lungs up to day 150	Khader et al. (158)
				1 log higher Mtb load in spleen at day 150		
B6 (6–12)	IL23 p19^−/−^	H37Rv (100 CFU), aerosol	No data	Day 120 and onward, 0.5–1 log higher Mtb load in lungs	Reduced no. of B-cell follicles at day 200 (Cxcl13 mediated)	Khader et al. (194)
					Strongly impaired IL-17, IL-22 production in lungs up to day 250	
B6 (6–12)	IL22^−/−^	H37Rv (100 CFU), aerosol	No data	No effect up to day 200	Suboptimal B-cell follicle development (Cxcl13-mediated)	Khader et al. (194)
B6 (6–9)	IL-17 RA^−/−^	H37Rv (1.10^3^ CFU), i.t.	Higher mortality (median survival: 18 vs. 35 weeks)	1.5 log higher Mtb load at week 12 and week 20 in lungs	Impaired cell recruitment (PMN, lymphocytes, Mo/DC)	Freches et al. (195)
					Increased IL-1β	
					Decreased TNF-α, IL-6 and IL-10	
B6 (6–12)	IL-17 RA^−/−^	H37Rv (100 CFU), aerosol	No data	No effect up to day 200	Suboptimal B-cell follicle development (Cxcl13-mediated)	Khader et al. (194)
B6 (8–12)	IL-17^−/−^	H37Rv (1.10^3^ CFU), i.t.	No data	1.5 log higher Mtb load	Reduced no. of granulomas at day 28	Okamoto Yoshida et al. (196)
B6 (6–8)	IL-17^−/−^	HN878 (100 CFU), aerosol	No data	1 log higher Mtb load in lungs at day 30 0.5 log higher Mtb load in lungs at day 60	Infection with HN878 showed robust production of IL-1β through TLR2, which supported increased IL-17 production compared to H37Rv and CDC1551	Gopal et al. (197)
B6 (6–8)	IL-17^−/−^	H37Rv, CDC1551 (100 CFU), aerosol	No data	No difference at day 30 and day 60		Gopal et al. (197)
B6 (8–12)	IL-17^−/−^	H37Rv (1.10^3^ CFU), i.t.	Higher mortality	1.5 log higher Mtb loads in lungs at day 30, 1 log higher Mtb loads at day 60 and day 120	Impaired granuloma formation, γδ T-cells primary source of IL-17	Umemura et al. (198)

*Red text indicates a harmful effect to the host; CFU, colony forming units; IL-17RA, IL-17 receptor A; i.t., intratracheal instillation*.

**Figure 4:**
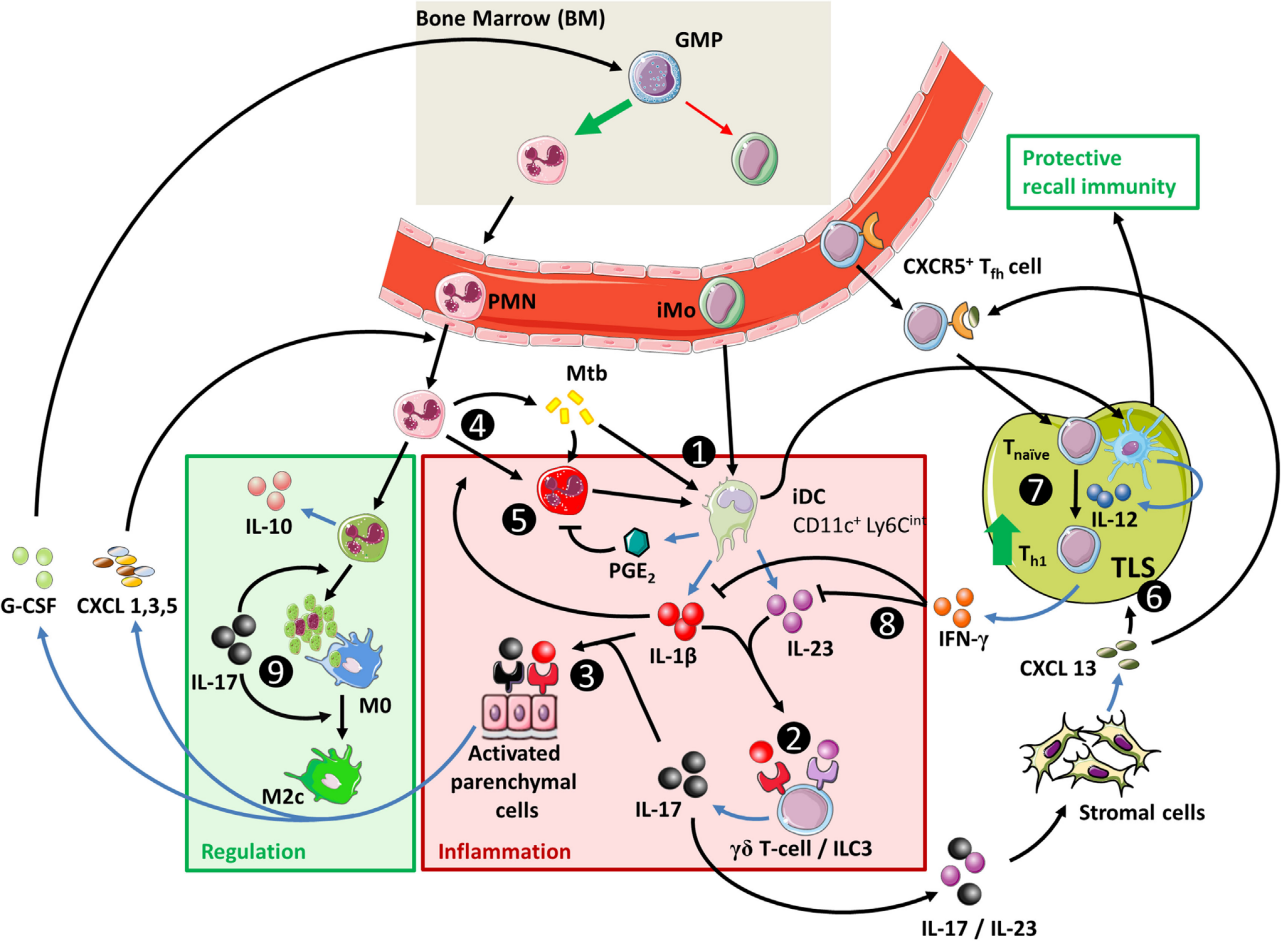
**The IL-23/IL-17 axis in acute tuberculosis**. (1) When inflammatory dendritic cells (iDC) recognize Mtb through membrane-bound toll-like receptors, they can secrete IL-1β, IL-23, and prostaglandin E2 (PGE_2_) (see Figure 1). This occurs more efficiently if iDC are activated through contact with Mtb-infected PMN, which also stimulates their migratory capacity to tertiary lymphoid structures (TLS) and promotes recall immunity (202–206). (2) The combination of IL-1β and IL-23 induces IL-17 production by γδ T-cells and possibly ILC3 (27, 117). (3) Activation of parenchymal cells by IL-17 in combination with IL-1β or other inflammatory mediators ultimately results in PMN influx. (4) PMN contribute to inflammation when stimulated by extracellular Mtb or inflammatory cytokines. (5) Activated PMN readily cause tissue damage through production of ROS and proteases; this effect is suppressed by activated iDC in a PGE_2_-dependent way (112, 207, 208). (6) IL-23 and IL-17 stimulate the local production of CXCL13 by stromal cells (194, 199). This promotes TLS formation and follicular helper T-cell migration to the site of infection. (7) CD40 ligation in the interaction between (i)DC and CD4^+^ T-cells is a strong stimulus for IL-12 production over IL-23 (Box 3) and leads to Th1 formation and IFN-γ production. (8) IFN-γ inhibits IL-1β production and shifts IL-23 production to IL-12, thus inhibiting IL-17 production and reinforcing the Th1 response (209). (9) In the absence of inflammatory stimuli, PMN can produce IL-10 and undergo apoptosis. Phagocytosis of apoptotic PMN induces an IL-10-producing regulatory M2c phenotype in macrophages and further contributes to resolution of inflammation.

Interestingly, these late protective effects result from effects induced during the initial phase infection (142, 195). This is due to the essential roles of IL-23 and IL-17 in the local formation of tertiary lymphoid structures (TLS) (199, 200). These structures are formed during early infection but can persist for longer periods of time and are associated with protective immunity in Mtb-infected mice (199, 201) (Table 4; Figure 4). Furthermore, IL-17 and IL-23 increase the expression of the chemokine CXCL13 (194, 197). This chemokine stimulates the influx of TLS-associated CXCR5^+^ follicular helper (T_fh_)-cell, which facilitate optimal localization of effector T-cell populations within the lung parenchyma, thereby promoting efficient T-cell-dependent macrophage activation and intracellular Mtb killing (194, 201).

On account of their ability to induce TLS formation, boosting IL-23 and IL-17 production is also an interesting strategy for vaccine-induced protection against TB. In this regard, IL-17 production by Th17 cells during recall responses is indeed dependent on IL-23 and could reduce mycobacterial loads in the lungs of Mtb-infected mice (210). Th17 cells preferentially migrate to the lungs and are better contained in the lungs compared to Th1 cells upon adoptive transfer to naïve mice (210, 211). The developmental flexibility of Th17 cells is illustrated in experiments where Mtb-antigen-primed Th17 cells have been adoptively transferred to naïve mice (210). Initially, these Th17 cells produce IL-17. However, upon recall immunity against Mtb, they primarily produce IFN-γ, with or without IL-17. Paradoxically, the latter switch results in a less effective reduction in bacterial loads compared to IL-17-producing Th17 cells that are adoptively transferred from IFN-γ^−/−^ mice.

This tendency of Th17 cells to produce IFN-γ instead of IL-17 during recall responses might explain the observation that IL-17 production during later phases of Mtb infection is dominated by γδ T-cells rather than CD4^+^ cells (117, 198).

When taken together, initial shaping of the local inflammatory environment by IL-17 and IL-23 during acute infection stimulates local TLS formation. This facilitates the development of more robust Th1 responses by improving contact between antigen-presenting cells (APC) and lymphoid cells (Figure 4). Furthermore, Th17 cells confer protective immunity during recall responses by their enhanced capacity to migrate to the lungs and stimulate T_fh_ responses compared to other CD4^+^ T-helper cell populations.

### 3.4 The Th17 Response, PMN, and Inflammatory Damage

IL-17 stimulates granulopoiesis in the bone marrow and increases PMN influx to the site of infection by inducing G-CSF, CXCL1, CXCL3, and CXCL5 expression by parenchymal cells in mice or G-CSF and IL-8 in humans (159). These effects of IL-17 are markedly enhanced through synergistic activation by inflammatory mediators such as IL-1β, TNF-α, or GM-CSF (164, 212, 213). In this regard, IL-17 is not a strong inducer of inflammation by itself, but rather amplifies preexisting inflammation. This IL-17-mediated “inflammatory boost” can positively shape adaptive immunity, but prolonged or repeated antigen exposure can also lead to PMN-mediated pathological inflammation (214). Since IL-17 signaling is inevitably linked to PMN influx, the role of PMN in TB provides an additional perspective on the effects of IL-17 signaling in TB.

Review of available literature on the role of PMN in TB yields a complex picture with seemingly conflicting effects (14, 166, 215). In patients with active TB, PMN are the predominantly infected cells in the airways and provide a permissive site for a burst of active mycobacterial replication prior to transmission (216). On the other hand, PMN from healthy individuals, especially when stimulated with TNF-α, show a strong bactericidal effect (217). In preclinical TB models, highly susceptible mouse strains such as I/St, CBA/J, or DBA/2 show an enhanced influx of apoptosis-resistant, highly phagocytic neutrophils that negatively affect survival compared to more TB-resistant C57BL/6 and BALB/c mice (218–220). Moreover, PMN are poor producers of essential cytokines such as IL-1α/β and IL-12p40 in the anti-TB response (90, 221). These effects in preclinical models primarily suggest a negative contribution of PMN to acute disease. However, increasing evidence suggests a supportive role for PMN in protective immunity. PMN can indirectly augment IL-1β-mediated inflammatory responses in macrophages after contact with Mtb (202, 204, 205). Also, and consistent with Th17 responses, PMN play an essential role in generation of protective recall responses in Mtb-infected mice (195, 206, 222). Early, but not late PMN recruitment is essential for IL-17-mediated long-term control of Mtb infection (195). This can be explained by the finding that DCs that acquire Mtb through uptake of infected PMN are better able to activate T-cells (203, 222). The importance of this mechanism is recently highlighted in Mtb-infected mice, showing that PMN-depletion during vaccination prevented the generation of specific Th1 and Th17 responses (206).

A second emerging protective role of PMN is their contribution to initiating inflammation resolution (223). In mouse TB models, PMN are the main producers of IL-10 in the lungs and can dampen inflammatory damage (224). In this regulatory role, PMN inhibit Th17 responses but do not interfere with IFN-γ-mediated Th1 immunity due to relative insensitivity of Th1 cells to IL-10 (224, 225). Another regulatory effect of PMN concerns their apoptosis and subsequent phagocytosis by macrophages in the absence of extracellular Mtb. This inhibits IL-23 production by these macrophages and induces a regulatory IL-10^high^ M2c phenotype under influence of IL-17 and IL-10 (see Figure 4) (226, 227). IL-17 can further contribute to this process by attenuating the anti-apoptotic effect of GM-CSF on PMN and by stimulating PMN apoptosis (228, 229).

Taken together, PMN recruitment to the site of infection is largely dependent on IL-17, but only in synergy with innate inflammatory cytokines such as IL-1β. Locally, these recruited PMN contribute to inflammation if pathogens are still present, improve dendritic cell function, and contribute to the formation of recall responses, or initiate resolution of inflammation in the absence of inflammatory or microbial stimuli.

### 3.5 Summary: The Role of Th17 Immunity in TB

The roles of IL-23 and IL-17 in TB are more subtle than the effects of Th1-related cytokines or T1-IFNs. Patient data are mostly limited to studies in PBMC. These show inconclusive results that are possibly confounded by the dynamics and heterogeneity of the Th17 response, which can range from highly pro-inflammatory IFN-γ/GM-CSF-producing Th17.1 cells to IL-10-producing regulatory Th17 cells.

Preclinical mouse TB models provide evidence for a protective role of the Th17 cytokines IL-23 and IL-17 in TB. These protective effects become apparent in the chronic phase of infection but result from IL-23/IL-17-mediated effects in the earlier, acute phase of infection. This is associated with early protective effects of IL-1β, which is a strong inducer of IL-23 and IL-17 (Figure 4). Mechanistically, evidence for the protective effects of IL-17 and IL-23 primarily points toward their role in the development of TLS during the acute phase of disease, which provides protective effects during later stages (199, 230). Additionally, IL-23 and IL-17 induce CXCL13 expression that mediates the influx of TLS-associated T_fh_ cells. TLS and T_fh_ responses facilitate optimal interactions between adaptive and innate immunity, contribute to granuloma formation, and improve the quality of T-cell recall responses in TB (201). In TB patients, TLS have also been associated with immune control, but more in-depth research is needed to establish their exact functional role and contribution to protective immunity (201).

Next to TLS formation and function, IL-23 and IL-17 mediate the influx of PMN into the lungs and the contribution of these cells to protective immunity in TB is increasingly recognized (206). Early, but not late PMN recruitment is essential for IL-17-mediated long-term control of Mtb infection (195) and DCs that acquire Mtb through uptake of infected PMN are better able to activate T-cells than DCs that directly interact with Mtb themselves (203, 222). The ability of IL-17 to induce the production of PMN-attracting chemokines in parenchymal cells is markedly improved when IL-17 signals in synergy with inflammatory mediators such as IL-1β, which again indicates synergy between IL-1β and IL-17 responses during acute TB. Prolonged activation of IL-1β and IL-17 responses can lead to massive accumulation of PMN, and their local necrotic death can also be damaging to the host. However, in the absence of inflammatory stimuli, PMN are an important source of IL-10 in the lungs and can initiate resolution of inflammation (Figure 4).

## 4 T1-IFNs, the Th17 Response and Their Interactions in Autoimmune Disease

Autoimmune diseases comprise a wide range of organ-specific and systemic disorders. Most systemic AID are considered classical B cell-mediated diseases, typified by circulating autoreactive antibodies against intracellular self-antigens. The clinical presentation of different AID varies, but evidence from genome-wide association studies points toward common immunogenetic mechanisms, as many systemic AID share disease-associated genes (231). Another trait particularly shared amongst different antibody-driven AID is the expression of a T1-IFN signature in both blood- and disease-affected tissue (232–234), the strength of which generally correlates with disease activity and severity (235–238). *Vice versa*, T1-IFN immunotherapy as treatment for other diseases is known to cause symptoms similar to those observed in AID, such as development of psoriatic lesions in MS or hepatitis C-infected patients (239, 240).

T-cells also have a major impact on the development and progression of AID and increasing evidence points toward crucial involvement of the Th17 response in the pathogenesis of multiple AID (160, 241). Th17 cells have been shown to be critical in the pathogenesis of MS and rheumatoid arthritis (RA) (19, 160). However, Th17 cells have also been associated with disease severity in AID characterized by a T1-IFN signature, such as systemic lupus erythematosus (SLE) (20, 233, 242, 243). Since T1-IFN signatures and Th17 responses are both associated with disease in AID, the question arises whether these two pathways act in concert to sustain and amplify autoimmune responses, or control each other (20, 21, 244). Therefore, we will discuss below the involvement of the T1-IFN and Th17 responses in AID individually as well as their interaction. We refer readers who are familiar with the contributions of T1-IFN and Th17 in AID to continue at Section 4.3 where we discuss the interaction between these pathways.

### 4.1 The Contribution of T1-IFNs to the Pathogenesis of AID

Most insight into the role of T1-IFNs in the pathogenesis of AID has been obtained in SLE, which was the first disease in which a T1-IFN transcriptional signature was identified in 2003 (235). Since then it has become clear that 60–80% of adult SLE patients and nearly 100% of pediatric SLE patients express a T1-IFN signature in their blood (245). Several mechanisms through which T1-IFNs contribute to disease in SLE, outlined below, have been elucidated.

#### 4.1.1 Induction of T1-IFNs in AID

Specifically IFN-α appears to play a central role in SLE pathogenesis (245, 246). As mentioned in Section 2.3 IFN-α is produced in an IRF7-dependent way by pDC and other myeloid cell types. In accordance, pDC have been found to be a major source of T1-IFNs in SLE (247, 248). Immune complexes (IC), consisting of antibodies bound to self-DNA, are a major trigger for IFN-α production by pDC in AID (249). However, pDC are not activated by self-DNA under steady state conditions, which indicates that additional stimuli are required. One such stimulus is the PMN-derived antimicrobial peptide LL37 (249), which convert inert self-DNA into a potent activator of endosomal TLR9 (250). Another stimulus is the nuclear protein high mobility group box 1 (HMGB1) protein, which is secreted by activated myeloid cells and passively released by necrotic, but not apoptotic cells (251). HMGB1 binds DNA, and the formed complexes bind with high affinity to receptor for advanced glycation end-products, which facilitates internalization into the endosome where TLR9 can be activated (249). Extracellular HMGB1 also triggers the recruitment of PMN and stimulates their formation of neutrophil extracellular traps (NETs) (252). NETs contain large amounts of nucleic acids and LL37 and are also a major driving factor behind chronic pDC activation and IFN-α production in SLE (253).

It deserves mention that NET formation is driven by reactive oxygen species (ROS), which in PMN are particularly produced by NADPH oxidase and subsequently processed by myeloperoxidase (254). Paradoxically, despite the capacity of NETs to induce T1-IFNs and the pathogenic role of T1-IFNs in SLE, NADPH oxidase appears to be protective in SLE (255). Lupus-prone mice deficient in NADPH-oxidase develop more severe SLE (255). Moreover, autoimmunity with T1-IFN signatures can still develop in individuals with chronic granulomatous disease, who lack NADPH-oxidase activity (256). This seeming contradiction has been partially explained by the observation that IgG autoantibody-mediated NETosis, which is most relevant in SLE, is specifically reliant on mitochondrial ROS, while NETosis induced by, e.g., TLR4 signaling is NADPH dependent (256). In line with this, NETs from SLE patients have been shown to contain mitochondrial DNA (256). Thus, the way NETs are induced, and the type of DNA that is present on NETs probably also influences their ability to induce T1-IFNs and their role in disease.

Taken together, TLR9-mediated IFN-α production by pDC in response to IC and NETs appears the major driving factor behind T1-IFN production in autoantibody-mediated AID. Additionally, the way NETs are induced and the type of DNA present on NETs can influence disease outcomes.

#### 4.1.2 Disease-Promoting Effects of T1-IFN in AID

Type I interferons exert a detrimental effect in AID through different pathways. In monocyte-derived cells, T1-IFNs stimulate maturation, increase phagocytic capacities (257), and increase the expression of co-stimulatory molecules (258). Also, T1-IFNs have a direct stimulating effect on T-cells. Together, these effects promote the generation of autoreactive T-cells, which support autoreactive B-cell responses (257, 259).

At cytokine level, T1-IFNs can induce the production of B-cell activating factor (BAFF) by myeloid cells (238, 260, 261). BAFF induction confers a significant proportion of T1-IFN-mediated damage in SLE as supported by the observation that IFN-α administration induces disease in SLE-prone mice but fails to do so in B-cell-deficient and BAFF-deficient mice on the same background (262). BAFF plays a central role in the development and selection of autoreactive B-cells (260). In line with this, increased BAFF expression correlates with disease severity in SLE (21, 260, 263). BAFF also induces class switch recombination in B-cells, leading to preferential expression of IgG and IgA over IgM, which is important for Fc-receptor-mediated NETosis induction in PMN (264). The clinical relevance of BAFF in SLE pathogenesis is illustrated by the current use of belimumab, a monoclonal antibody against BAFF, as treatment for SLE (265). Interestingly, targeting BAFF is effective in SLE patients, while B-cell depleting therapies using CD-20-targeting rituximab show disappointing results in phase III clinical trials (266, 267). This suggests effector functions of BAFF other than B-cell activation. In this regard, BAFF can act as a co-stimulatory molecule for T-cells and promote Th17 development (268, 269). BAFF can also directly activate plasma cells, which are not depleted by rituximab (270, 271).

### 4.2 The Contribution of Th17 in the Pathogenesis of AID

#### 4.2.1 GM-CSF-Secreting Th17.1 Cells

Pathogenic effects of Th17-mediated immunity in AID have been studied most detailed in MS and RA and their respective mouse models, experimental autoimmune encephalitis (EAE), and collagen-induced arthritis (160, 242). MS was long believed to be primarily driven by an IL-12/Th1 response, but this concept was challenged by observations in the EAE mouse model for MS showing that the IL-23p19 subunit instead of IL-12p35 (see Box 3) caused disease (272). In addition, the classic cytokines of Th1 and Th17 immunity, i.e., IFN-γ and IL-17, respectively, were found dispensable in EAE and instead GM-CSF appeared to be the effector cytokine responsible for IL-23-induced encephalopathy (118). Notably, while most studies agree on a central pathogenic role for GM-CSF in MS, conflicting results are reported regarding its cellular source (19, 273–275). One study shows that GM-CSF expression in MS patients is promoted by the IL-12/T-bet/Th1 axis, instead of IL-23 as observed in mouse EAE (273). Other publications report that B-cells are a major source of GM-CSF and specifically act in concert with Th17 cells (274, 276). In accord with these discrepant results, MS is shown to be a heterogeneous disease that can be driven by either Th1 or Th17 immunity (242), which also has implications for therapy as will be discussed in Section 4.3.1.

One interesting observation in this regard is the development of “hybrid” Th17.1 cells that express markers of both Th17 cells and Th1 cells. Naïve CD4^+^ T-cells in both mice and man do not express the IL-23 receptor and can either differentiate into T-bet^+^ Th1 cells under influence of IL-12 or differentiate into CCR6^+^ Th17 cells under influence of IL-6 and TGF-β (161). These IL-6/TGF-β-differentiated Th17 cells have low inflammatory potential and are prone to adopt an IL-10-producing regulatory phenotype. However, IL-6 also induces STAT3-dependent upregulation of IL-23 receptor (277). Subsequent (re)activation of such IL-6-primed Th17 cells by IL-23 increases Th1-associated T-bet expression and generates inflammatory IFN-γ/GM-CSF-producing Th17.1 cells (161). These cells can also switch their chemokine receptor profile and become CCR2^+^ instead of CCR6^+^ (161). Expression of CCR2 by Th17.1 cells can contribute to their inflammatory potential as it can divert their migration to sites without concomitant influx of regulatory T-cells, which depend on CCR6 for their migration (278).

Mechanistically, it was shown in a mouse EAE model that GM-CSF exerted its pathogenic effector function by stimulating IL-1β production by monocyte-derived cells (279). This suggests a positive inflammatory feedback loop, since IL-1β in turn promotes IL-23 production and development of Th17.1 cells (118). A similar pathogenic Th17.1 response is observed in RA, which was the first AID in which IL-1β inhibition was approved for clinical use (280). Also, regarding the distinction between Th17 and Th17.1 responses in RA, it should be noted that anti-GM-CSF therapy shows more promise than anti-IL-17 in clinical phase I/II trials (160, 281).

#### 4.2.2 The Contribution of IL-17-Producing Th17 Cells to AID Pathogenesis

Next to GM-CSF-secreting Th17.1 cells, regular IL-17-producing Th17 cells also have been identified as pathogenic in other AID. This is best exemplified by the clinical successes of targeting IL-17 in psoriasis (282). Th17-associated pathogenic effects in SLE also appear to be driven by IL-17 rather than GM-CSF (21, 283). This is further supported by the specific contribution of PMN to disease in SLE, which is dependent on IL-17, opposed to GM-CSF that primarily influences the inflammatory potential of monocytes in MS and RA.

### 4.3 Interactions between T1-IFNs and the Th17 Response in AID

**Figure 5:**
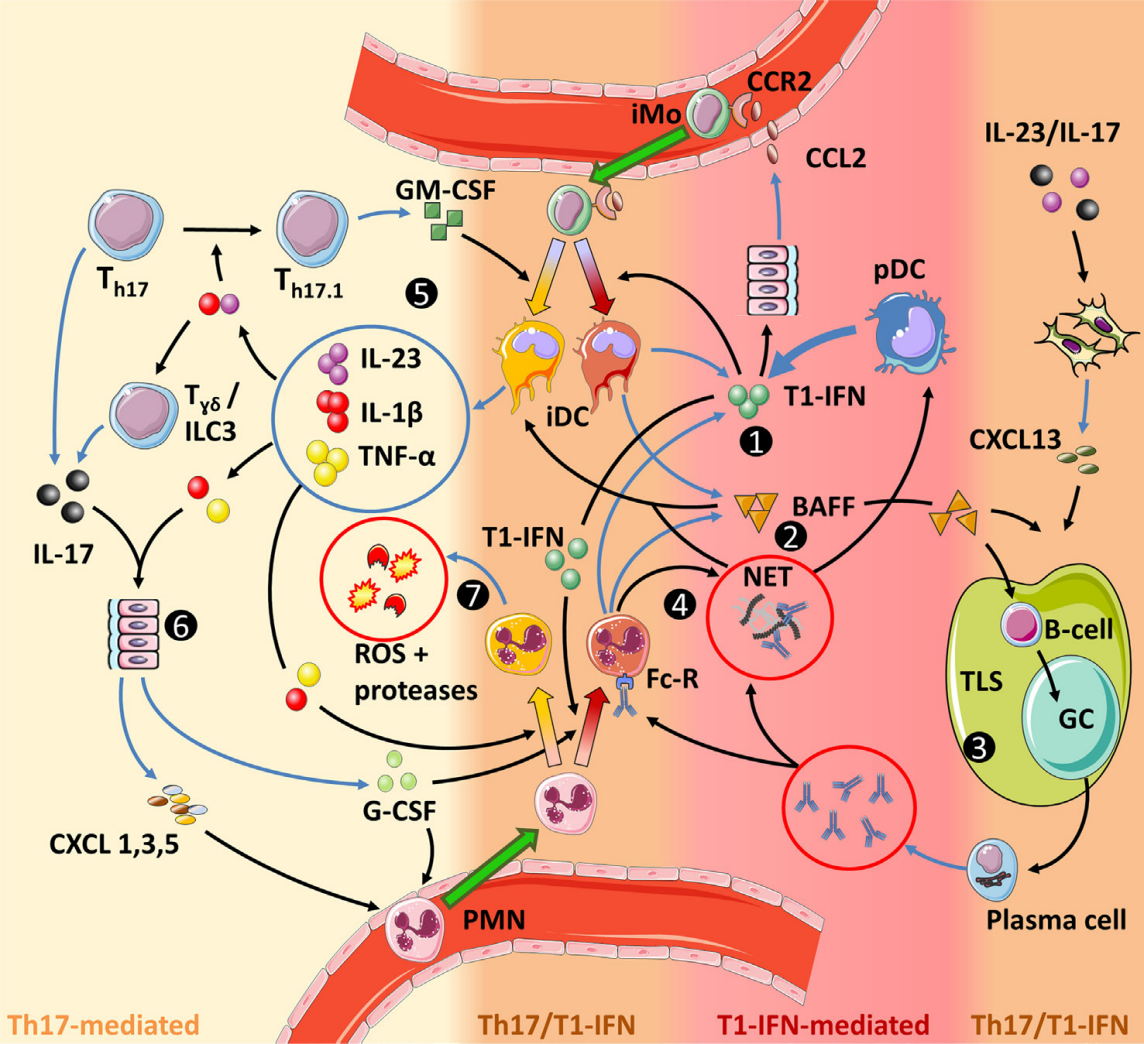
**Interactions between type I interferons (T1-IFNs) and Th17 immunity in autoimmune diseases**. The color grading in the figure indicates the level of involvement of either Th17 immunity or T1-IFN-associated signaling. (1) T1-IFNs, primarily produced by plasmacytoid dendritic (pDC) but also by inflammatory dendritic cells (iDC) and PMN, prime the latter cells toward a T1-IFN/B-cell activating factor (BAFF)-producing phenotype, promote NETosis by PMN and stimulate monocyte migration by inducing CCL2 production. (2) BAFF activates B-cells, stimulates tertiary lymphoid structures (TLS) formation together with CXCL13, directly promotes Th17 differentiation (not shown), and stimulates the release of IL-1β by iDC. (3) TLS facilitate optimal interaction between activated B-cells and antigen-presenting cells (APC), while necrosis, neutrophil extracellular traps, and T1-IFN increase the chance that these APC present self-antigens. Subsequent germinal center (GC) reactions within these TLS result in B-cells differentiating into plasma cells that produce large quantities of autoantibodies. These autoantibodies can mediate tissue damage and sustain a self-amplifying loop by inducing NETosis through binding the Fc-receptor on PMN. B-cells can also contribute to Th17 immunity by their ability to secrete IL-6 and GM-CSF (not shown and uncertain if this is BAFF dependent). (4) NETs trap antibodies. This facilitates their Fc-receptor-mediated internalization by pDC in which they stimulate T1-IFN production through endosomal TLR9 activation. Circulating NETs also stimulate IL-1β production by iDC and can mediate tissue damage. (5) In a pro-inflammatory feedback loop, IL-23 stimulates the development of GM-CSF-producing Th17 cells (Th17.1), which in turn, together with BAFF and/or NETs stimulate an inflammatory phenotype in iDC. (6) IL-1β and IL-23 stimulate IL-17 production by γδ T-cells, while concomitant stimulation with IL-1β and TNF-α is required for IL-17-induced G-CSF and chemokine production in parenchymal cells. (7) IL-1β and TNF-α activate PMN to release reactive oxygen species (ROS) and proteases that cause tissue damage. Furthermore, GM-CSF increases longevity of PMN (not shown). Finally, the priming of PMN and monocytes prior to entering the site of disease is important for their eventual effector function. For monocytes this is shown in more detail in Figure 3.

Systemic lupus erythematosus and other autoantibody-mediated AID show a pathogenic role for T1-IFNs, while T-cell-mediated AID, such as MS, are driven primarily by GM-CSF-stimulated IL-1β production. With the functional dichotomy of IL-1β and T1-IFNs in mind, as shown in Figure 2, MS and SLE seem to be opposite ends of the disease spectrum in AID instead of demonstrating interactions between T1-IFNs and the Th17 response. However, the existence of different Th17 subsets might explain this seeming disparity and suggest roles for GM-CSF-producing Th17.1 cells in MS and regular IL-17-producing Th17 cells in SLE. Both Th17 responses interact differently with T1-IFNs as will be discussed here. We identify three relevant interactions: (1) Th17.1 responses are fueled by T1-IFN-stimulated influx of CCR2^+^ inflammatory monocytes; (2) a pathological IL-17/T1-IFN/BAFF axis driven by NET-forming PMN; and (3) Th17 immunity and T1-IFNs collaborate in the generation and function of TLS. An overview of these pathways is presented in Figure 5.

#### 4.3.1 T1-IFNs Can Contribute to Th17.1-Mediated AID

Among MS patients treated with IFN-β, approximately 30–50% do not respond favorably to treatment (284). It was shown that IFN-β suppresses Th1-mediated inflammation in MS but is ineffective and may even exacerbate Th17-mediated inflammation (19). This is one of the first studies that report a detrimental interaction between T1-IFNs and Th17 responses. Given the importance of Th17.1 cells in MS, this negative outcome might be explained by the observation that IFN-β therapy in MS increases CCL2 production (285). Expression of this chemokine in the brain recruits inflammatory CCR2^+^ monocytes as well as Th17.1 cells, which switch their chemokine receptor profile from CCR6^+^ to CCR2^+^ upon terminal differentiation (161). Moreover, Th17.1 cells stimulate IL-1β production in CCR2^+^ monocytes (279, 286). Inflammatory monocytes may differentiate locally into dendritic cells further stimulating Th17 responses (287). Thus, a strongly pro-inflammatory condition is created in Th17.1-mediated MS. Since regulatory T lymphocytes rely on CCR6 rather than CCR2 (279), recruitment of these anti-inflammatory cells does not appear to hold pace with the influx of inflammatory monocytes and Th17.1 cells in MS.

#### 4.3.2 A Pathological IL-17/T1-IFNs/BAFF Axis in AID

IL-17 induces PMN influx through induction of G-CSF and chemokines (see Section 3.4), which contribute to the production of IFN-α by pDC *via* the NETosis process (see Section 4.1.1). However, increasing evidence suggests a more prominent contribution of IL-17 and PMN to T1-IFN-mediated disease in SLE. First, besides being major inducers of IFN-α production by pDC upon NETosis, PMN also appear to be a significant source of IFN-α themselves (288, 289). This was related to their sheer numbers, as circulating pDC were 27 times more efficient in secreting IFN-α, but PMN were 100 times more frequent (289). Second, both T1-IFNs and IL-17-induced G-CSF prime PMN for NETosis (250, 290). In accord, circulating PMN of SLE patients are also the main cells expressing the transcriptional T1-IFN signature and release more NETs than PMN from healthy individuals (250, 253, 288, 289, 291, 292). Thirdly, T1-IFNs stimulate BAFF production, which is essential for T1-IFN-mediated pathogenic effects in mouse SLE (261, 262, 293). It is recently shown that IL-17 also induces BAFF production and that IL-17-driven, G-CSF-dependent PMN recruitment drives plasma cell responses during emergency granulopoiesis in a BAFF-dependent way (271). Also, therapeutically administered G-CSF, which is physiologically induced by IL-17, increases BAFF production by PMN (294).

These interactions indicate a prominent role for IL-17-mediated PMN influx in T1-IFN-production and induction in AID and synergistic induction of BAFF production by IL-17 and T1-IFNs. In support of this, IL-17 and Th17 cells are associated with disease severity in SLE to similar extent as T1-IFNs (20, 21, 241, 244). In turn, BAFF can promote Th17 responses (268, 269). This further suggests an inflammatory loop with a central role for PMN in which IL-17, T1-IFNs, and BAFF continuously increase each other’s production and contribute to autoantibody-mediated responses.

#### 4.3.3 T1-IFNs, Th17 Responses, and TLS in AID

Finally, T1-IFNs and Th17 responses converge onto the development and functioning of TLS. In these structures, T_fh_ cells support germinal center (GC) reactions in which B-cells differentiate into antibody-producing plasma cells and memory cells (295). As expected from their function, TLS and T_fh_ cells are essential components in the pathogenesis of multiple autoantibody-mediated AID (296–303). The cytokines IL-17 and IL-22 secreted by ILC3, γδ T-cells and Th17 cells are required for local TLS formation (199, 230, 304). T1-IFN- and IL-17-induced BAFF promote the formation and integrity of GCs within TLS and stimulate T_fh_ development (305, 306). T1-IFNs directly induce the expression of the T_fh_-markers CXCR5 and PD-1 on T cells (307, 308). Also, T1-IFNs promote the survival of aberrantly selected B-cells in the GC reactions during SLE directly and indirectly through BAFF induction as discussed in Section 4.2.2. Thus, it appears that by stimulating TLS development, the Th17 response facilitates an environment that promotes selection of autoreactive B-cells under influence of T1-IFNs and BAFF.

Taken together, several lines of evidence exist for interactions between the Th17 response and T1-IFNs in systemic AID. Current data support a scenario in which Th17 immunity fuels T1-IFN-related pathology by mediating PMN influx and driving TLS formation, which facilitates T1-IFN/BAFF-mediated plasma cell responses and autoantibody production. In turn, T1-IFNs can support pathogenic Th17.1 responses in AID by driving the influx of CCR2^+^ inflammatory monocytes and potentially CCR2^+^ Th17.1 cells themselves, which locally drive IL-1β mediated inflammation. An overview of these interacting pathways is shown in Figure 5.

## 5 Interactions Between T1-IFNs and Th17 Immunity in TB

In the previous section, we have outlined how T1-IFNs and Th17 immunity interact in AID (illustrated in Figure 5). These interactions primarily concern (1) Th17.1 responses fueled by T1-IFN-stimulated influx of CCR2^+^ monocytes; (2) The IL-17/T1-IFNs/BAFF axis driven by NET-forming PMN; and (3) synergism between Th17 immunity and T1-IFNs in TLS formation and function. In this section, we assess the relevance of these three pathways in TB based on cell types and effector molecules involved. Each subsection contains a part of Figure 5, supplemented with relevant finding and outstanding questions in TB.

### 5.1 Th17.1 Responses in TB

**Figure 6:**
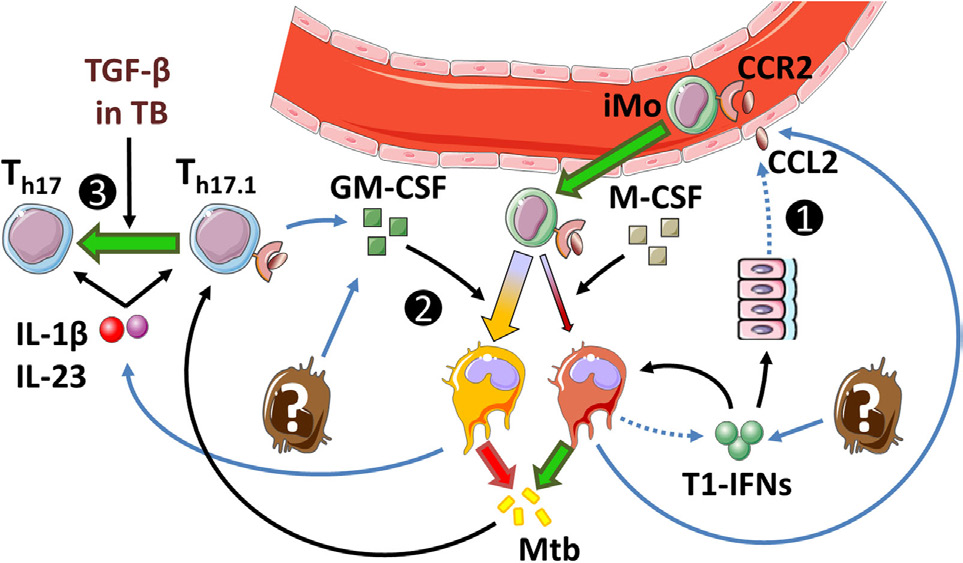
**Th17.1 responses in tuberculosis (TB)**. (1) Type I interferons (T1-IFNs) induce CCL2 production in parenchymal cells and MDM, but not GMDM. This induces the influx of CCR2^+^ monocytes that mediate detrimental effects in TB as Mtb-permissive cells develop upon T1-IFN stimulation. (2) GM-CSF increases IL-1β production, limits responsiveness to T1-IFNs, and increases Mtb-killing potential. However, the exact cellular source of GM-CSF in TB is unknown. (3) Patients with active TB overexpress TGF-β, which may drive Th17 development over Th17.1 in the presence of IL-1β and IL-23. Dotted lines implicate mechanisms shown in autoimmune diseases that have not been confirmed in TB. Outstanding questions: (1) What is (are) the cellular source(s) of T1-IFNs in TB? (2) What is the ratio between different Th17 subsets in TB? (3) Do T-cells contribute to GM-CSF production in TB?

Studies in MS and RA emphasize the difference between GM-CSF/IFN-γ-producing Th17.1 cells and regular IL-17-producing Th17 cells. The former primarily increase the inflammatory potential of monocytes (Figure 6), while the latter are more closely associated with PMN. Data on subtypes of Th17 cells and particularly Th17.1 cells in human TB are limited. One study shows that circulating GM-CSF^+^ T-cells are not increased in ATB compared to LTBI, but it is unclear if this concerns Th17.1 cells or Th1 cells (309). Interestingly, GM-CSF production by both granuloma-associated T-cells and circulating CD4^+^ T-cells in TB patients only occurs after mycobacterial antigen stimulation (309, 310). In mice, adoptively transferred Mtb-primed Th17 cells that produce IL-17 upon transfer, predominantly produce IFN-γ upon subsequent contact with Mtb, which is suggestive of a Th17.1 phenotype (210).

Th17.1 cells in AID result from prolonged innate IL-1β and IL-23 signaling. With regard to the role of IL-1β and IL-23 in human TB, IL-1β is essential for the expansion of both IFN-γ^−^IL-17^+^Th17 cells and IFN-γ^+^IL-17^+^Th17 cells (311, 312). IL-23 promotes the development of IFN-γ^+^IL-17^+^Th17 cells but promotes IFN-γ^−^IL-17^+^Th17 cells if TGF-β is concomitantly present (312). Since active TB is associated with elevated TGF-β levels (178, 313, 314), it is possible that Th17.1 cell differentiation does not play a major role, but this remains to be demonstrated.

Th17.1-derived GM-CSF exerts a pathogenic effect in AID by stimulating IL-1β production in CCR2^+^ monocytes. Although the role of Th17.1 cells in TB is uncertain, other cells such as NK cells and Th1 cells can also produce GM-CSF in TB, and during the course of infection, GM-CSF levels progressively increase in the lungs of Mtb-infected mice (125, 315). The functional role of GM-CSF is of interest in TB, because it importantly impacts on CCR2^+^ monocytes, which play a central role in T1-IFN-mediated pathogenic effects. T1-IFNs stimulate the influx of inflammatory CCR2^+^ monocytes but inhibit their IL-1β production and stimulate their differentiation into Mtb-permissive cells (see Figure 3). In contrast, GM-CSF is protective during acute TB, which is in line with the protective effects of IL-1β in this phase of disease. Mice deficient in GM-CSF succumb rapidly to infection due to their inability to mount Th1 responses (316, 317). Transgenic mice that overexpress GM-CSF in the lungs but are GM-CSF-deficient in all other organs can develop Th1 responses, but still succumb to infection more rapidly than wild-type mice due to their inability to develop a normal granulomatous response (316, 317). Evidence from *in vitro* studies suggests that GM-CSF exerts its protective effect in TB by countering the effects of T1-IFNs in CCR2^+^ monocytes (36, 94). Under physiological conditions, monocytes differentiate under influence of M-CSF into monocyte-derived macrophages (MDM). These MDM have a CCR2^low^ phenotype, readily produce CCL2 and IL-10 in response to T1-IFNs, and have a low Mtb-killing capacity (94, 156, 318, 319). Conversely, monocytes that differentiate under influence of GM-CSF (GMDM) are CCR2^high^, relatively unresponsive to T1-IFN signaling, produce small amounts of CCL2 and IL-10, and have better Mtb-killing capacities than MDM in response to activation by IFN-γ (36, 126).

The relative unresponsiveness of GMDM to T1-IFNs might explain why preclinical studies primarily show effects of T1-IFNs during acute TB when the GM-CSF/M-CSF ratio in the lungs is relatively low, but less pronounced effects during later stages when GM-CSF-levels progressively increase (see Section 2.4.3; Figure 3) (125). However, similar to IL-1β, prolonged GM-CSF signaling also appears detrimental in TB. In particular, GM-CSF contributes to foamy macrophage development during later stages of infection, which can sustain persistent mycobacteria and contribute to inflammation (125, 320).

In summary, relatively few data are available on Th17.1 cells or T-cell-derived GM-CSF in TB. The requirement for antigen stimulation of T-cells to induce expression of GM-CSF is interesting. However, elevated TGF-β levels in TB patients suggest a limited contribution of Th17.1 cells to disease, as TGF-β favors the development of regular IL-17-producing Th17 cells. Regardless of its cellular source, preclinical TB studies support a protective role for GM-CSF during acute infection. GM-CSF causes monocytes to differentiate into cells with decreased T1-IFN responsiveness and increased Mtb-killing potential compared to their M-CSF-differentiated counterparts. However, during chronic Mtb infection, high GM-CSF levels appear detrimental as they stimulate foamy macrophage development and inflammation.

### 5.2 The IL-17/T1-IFNs/BAFF Axis in TB

**Figure 7:**
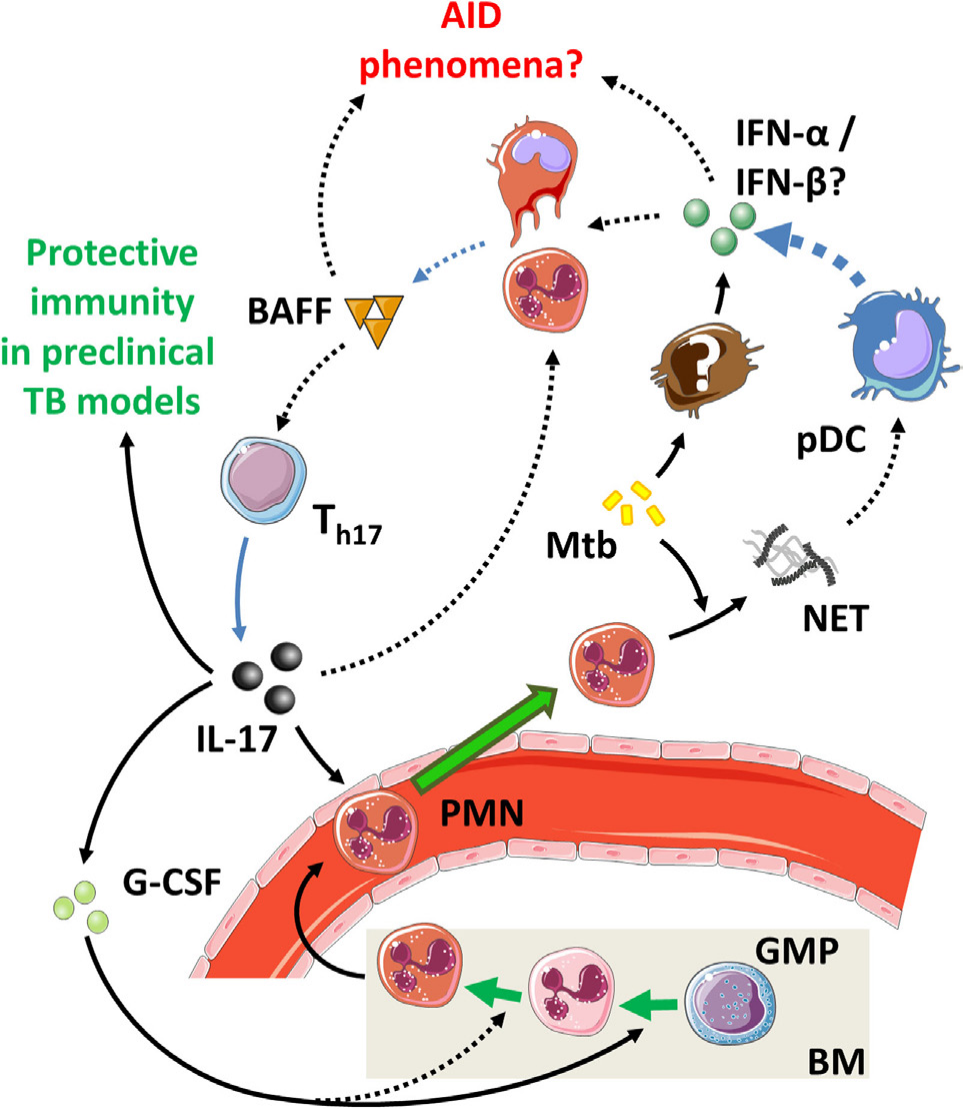
**The IL-17/type I interferons (T1-IFNs)/B-cell activating factor (BAFF) axis in tuberculosis (TB)**. Mtb actively induces NET formation by PMN, but subsequent activation of IFN-α production by plasmacytoid dendritic (pDC) appears less relevant in TB than in autoimmune diseases (AID). IL-17 levels from TB patients vary (Table 3), but preclinical models support a protective role. Dotted lines implicate mechanisms present in AID that have not been confirmed in TB. Outstanding questions: (1) What are the specific contributions of IFN-α vs. IFN-β to disease in the different phases of TB? (2) Do BAFF and T1-IFNs promote the observed autoimmune phenomena in TB?

In the previous paragraph, it was discussed that regular IL-17-producing T-cells are more likely to play a role in TB than Th17.1 cells. Opposed to Th17.1 cells, regular Th17 cells exert their effect primarily through PMN instead of CCR2^+^ monocytes in AID. Particularly in SLE, this was shown to be part of a pathogenic axis together with T1-IFNs and BAFF. The roles of T1-IFNs and IL-17 in TB have been discussed already in Sections 2 and 3. In this section, we assess the roles of the other components of the IL-17/T1-IFNs/BAFF axis in TB, which include PMN-derived NETs, pDC, and BAFF (Figure 7).

#### 5.2.1 PMN, NETs, and pDC in TB

PMN isolated from SLE patients are the primary cells that express the transcriptional T1-IFN signature. Furthermore, a specific subclass of PMN, termed low-density granulocytes (LDG) have been identified in SLE that express a pro-inflammatory phenotype, has increased T1-IFN-production and more readily form NETs than PMN from healthy individuals (291, 292). Similar to SLE, the transcriptional T1-IFN signature in TB patients is mostly expressed in PMN (58). Moreover, LDG are also present in TB patients and correlate with disease severity, but it is unclear if these cells also have a similarly increased tendency for NETosis as their SLE counterparts (321). Nevertheless, NETosis does occurs in TB, as Mtb readily induces NETosis itself in PMN in an ESX-1-dependent way and can even stimulate extracellular trap formation in macrophages (204, 322–324).

Neutrophil extracellular traps are strong inducers of IFN-α production in pDC in SLE (256). Conversely, pDC produce only small amounts of IFN-α and appear of minor clinical significance in TB (325). In accord, circulating pDC are elevated in SLE (326) but reduced in TB patients (327).

Final support for a limited role of pDC in TB pathogenesis comes from the observation that pDC produce IFN-α after endosomal TLR-activation, while it is shown that Mtb primarily induces IFN-β through activation of cytoplasmic PRRs (see Section 2.3) (81, 250). While both IFN-α and IFN-β signal through IFNAR, this diversification in cellular source and type of T1-IFN that is induced can have important consequences for TB pathogenesis (see Box 4).

Box 4 **IFN-α or IFN-β: which is relevant in tuberculosis (TB)?**

IFN-α and IFN-β both exert their effect by binding to IFN-α/β receptor, but increasing evidence from autoimmune diseases (AID) and viral infections suggests divergent effector functions (328, 329). In AID, this is illustrated by the pathogenicity of IFN-α in systemic lupus erythematosus (SLE) opposed to the therapeutic application of IFN-β as immunosuppressive treatment in multiple sclerosis (MS). Recently, these different immunoregulatory roles of IFN-α and IFN-β in SLE and MS have been confirmed by more detailed analysis of blood transcriptional profiles in patients (330). The molecular explanation for the differential function of IFN-α and IFN-β traces back to subtle differences in receptor binding, signaling cascades, and feedback mechanisms initiated and has been reviewed in detail elsewhere (28, 331).

The specific contributions of IFN-α and IFN-β to the host response in infectious disease have been studied particularly in mice infected with lymphocytic choriomeningitis virus. This work supports an immune-stimulating, antiviral role for IFN-α as opposed to an immunosuppressive effect by IFN-β (328, 331, 332). IFN-β specifically inhibits antiviral T-cell responses and promotes viral persistence (331). In contrast, IFN-α-signaling associates with tissue damage and antiviral activity (331, 332).

In TB, evidence for the involvement of both type I interferons is present. Reactivation of TB has been reported specifically after treatment of patients with IFN-α, but not IFN-β (50–57). Also, mice infected with virulent Mtb strains specifically show higher IFN-α levels in the lungs compared to less virulent strains (64, 65). However, IFN-α-producing plasmacytoid dendritic cells seem to be of minor significance in TB patients (325, 327), and preclinical studies show that Mtb preferentially induces IFN-β through cytoplasmic pattern recognition receptors and IRF3 instead of IFN-α through endosomal toll-like receptors and IRF7 (79–81). Mycobacterial persistence in patients with TB is a major clinical problem and in line with the immunosuppressed state in active TB primarily supports a role for IFN-β (178, 333). However, exaggerated innate responses are also observed in TB where IFN-α might be involved. This is supported by recent evidence showing that IRF7 drives excessive innate inflammation during bacterial infections and provides an interesting therapeutic target (334). Taken together, little is known about the separate effects of IFN-α and IFN-β in TB, but clinical and preclinical studies support a role for both in different disease contexts. The diversification of IFN-α and IFN-β responses in transcriptional signatures observed in AID patients and the distinct effects of IFN-α and IFN-β in experimental LMCV infection therefore provide highly interesting perspectives for TB.

#### 5.2.2 BAFF in TB

Both T1-IFNs and IL-17 can induce BAFF expression, which contributes to disease in SLE as illustrated by the clinical successes of BAFF-inhibition (261, 271, 335). BAFF increases B-cell numbers and antibody titers (293, 336) and treatment with anti-BAFF in SLE patients reduces serum IgG levels (335). The role of BAFF in TB has been explored to a much lesser extent, with currently one paper demonstrating BAFF levels to be elevated in patients with active TB without elaborating on its functional contribution to the host response (337).

The functional role of BAFF in TB might be of particular interest given its stimulation of humoral immunity and the recently demonstrated protective effects of antibody-mediated immunity in TB patients (338, 339). Next to antibody-mediated protection, B-cells also essentially support T-cell responses in TB, but circulating B-cells are dysfunctional and reduced in absolute numbers in patients with active TB (340). The protective effects of Mtb-specific antibodies and B cells in TB suggest that increased BAFF levels may supports host responses by stimulating antibody production and perhaps other B cell functions such as stimulating T cell responses (338). However, high BAFF levels also predispose for the development of autoreactive B-cells in AID (341). Thus, elevated BAFF levels in TB could relate to the observation that up to 32% of patients with active TB have elevated autoantibody levels (12). Such correlations between elevated BAFF levels and autoimmunity have been demonstrated in other chronic infections (342).

Additional support for a supposed protective role of BAFF in TB comes from its interaction with IL-17, which shows protective effects in preclinical early infection phase TB models, as discussed in Section 3. IL-17 stimulates the migration of PMN to lymphoid structures where they can produce large quantities of BAFF that directly drive plasma cell responses. Also, IL-17-induced G-CSF primes PMN for BAFF production upon activation (271, 294). *Vice versa*, elevated BAFF levels have been reported to increase Th17 immunity in AID and infection (268, 269, 343).

Taken together, preliminary pieces of evidence support the presence of interactions between IL-17, T1-IFNs, and BAFF in TB, similar to those demonstrated in AID. This primarily includes the presence of NETs and elevated BAFF levels. However, despite the T1-IFN signature observed in TB, NET-induced IFN-α production by pDC appears less relevant in TB than in AID, and the specific contributions of IFN-α and IFN-β are of high interest in TB, but currently largely unknown. Studies in TB patients show protective effects of antibody-mediated immunity but also elevated titers of autoantibodies. This supports a view in which BAFF is protective in TB, but excessive BAFF levels, driven by either T1-IFNs or IL-17 can also increase the chances of developing autoimmunity in TB patients.

### 5.3 TLS in TB

As a third place of interaction, IL-17, T1-IFNs, and BAFF converge in the local formation and functioning of TLS. In these structures, T_fh_ cells support GC reactions in which B-cells differentiate into plasma cells and memory cells (295). As discussed in Section 4.3.4, observations in AID suggest that Th17 responses drive TLS development and facilitate an environment that promotes development of autoreactive B-cells under influence of T1-IFNs and BAFF. Conversely, both TLS and T_fh_ cells are associated with immune control in TB patients and preclinical TB models, which is in line with the protective role of humoral immunity in TB discussed in the previous section (194, 201, 344, 345). Here, we discuss how TLS and T_fh_ responses are associated with protective immunity in TB and how interactions between IL-17, T1-IFNs, and BAFF may contribute to this immune response.

Migration of CXCR5^+^ T_fh_ cells into TLS is largely dependent on CXCL13, which is primarily induced by IL-23 and IL-17 in mouse TB models but can also be induced by T1-IFNs, as demonstrated in viral infections (194, 346). Mechanistically, CXCR5^+^ T_fh_ cells mediate their protective effect in Mtb-infected mice by facilitating optimal localization of effector T-cell populations within the lung parenchyma, thereby promoting efficient T-cell-dependent macrophage activation and intracellular Mtb killing (194, 201).

Another interesting observation regarding T_fh_ responses concerns the induction of PD-L1 expression on APC and PD1 on T-cells by T1-IFNs (43, 136, 307). In TB circulating PMN primarily express the T1-IFN signature but also overexpress PD-L1 (58, 347). The interaction of PD-L1 with PD1 on CD4^+^ T-cells is a key immunological checkpoint in TB that limits excessive T-helper responses (33, 35). In line with this, PD1-deficient mice are extraordinarily susceptible to TB (34). T_fh_ cells constitutively express PD1^+^, which distinguishes them from conventional CD4^+^ T_h_ cells. Interestingly, while increased PD1/PD-L1 interaction suppresses conventional T-helper responses, the opposite is observed for T_fh_ responses (348). Interaction between PD1^+^ T_fh_ cells with PD-L1 has a stronger suppressive effect on the regulatory subset of T_fh_ cells than on stimulatory T_fh_ cells and results in a net increase of T_fh_ activity (348).

Taken together, IL-17, T1-IFNs, and BAFF act in concert to drive TLS formation and T_fh_ responses. These responses support the development of autoreactive B-cells and the subsequent production of autoantibodies in AID but confer protective immunity in TB by improving the interaction between adaptive and innate cells and facilitating antibody production, while simultaneously inhibiting excessive inflammation by conventional CD4^+^ T-cell responses.

## 6 Concluding Remarks

The notion that complex mechanisms beyond Th1 immunity are at play in TB immunity is supported by (1) the unsatisfactory results of vaccine strategies aimed at boosting Th1 immunity in TB patients (31); (2) the inflammatory damage associated with increasing IFN-γ production by T-cells in the lungs of Mtb-infected mice (33); and (3) the host-detrimental effect of targeting the Th1-inhibiting PD1/PD-L1 interaction in mice (34, 35).

Patients with active TB express a T1-IFN transcriptional signature in their circulating leukocytes, but the exact identity and functional role of T1-IFNs in patients remains to be elucidated (62). Others have speculated that deleterious effects of T1-IFN-signaling during bacterial infections are tolerated because of their ability to suppress myeloid cell responses (41). This review highlights two additional aspects of T1-IFNs that are of interest in TB. The first concerns the preconditioning of myeloid cells prior to their contact with T1-IFNs. IFN-γ priming appears essential for the induction of an Mtb-permissive phenotype, and monocytes that differentiate under GM-CSF are less responsive to T1-IFNs than their M-CSF-differentiated counterparts (Figure 3) (43, 94). The second aspect is the diversification of IFN-α and IFN-β responses on a transcriptional and functional level as explained in Box 4. We propose that the inflammatory effects of IRF7-mediated IFN-α might contribute to excessive innate inflammatory responses in TB, while the immunosuppressive effects of IFN-β are more likely to support mycobacterial persistence.

Determination of the role of the Th17 response in TB is impeded by its heterogeneity, reflected in the presence of different Th17 subsets with ranging inflammatory potentials. Observations in AID emphasize the difference between IFN-γ/GM-CSF-producing Th17.1 cells and regular IL-17-producing Th17 cells. The exact role of T-cell-derived GM-CSF in TB remains to be determined, but preclinical TB studies show a protective role for GM-CSF on monocyte differentiation in the acute phase of TB. In contrast, IL-17 and PMN appear more relevant in chronic control of Mtb infection and recall immunity.

Immunological similarities between TB and AID may result from commonly activated pathogenic pathways. Alternatively, compensatory mechanisms induced by one disease might predispose for the development of the other. Interactions between IL-17, T1-IFNs, and BAFF form a pathological axis in AID that promote autoantibody-mediated autoimmunity.

**Figure 8:**
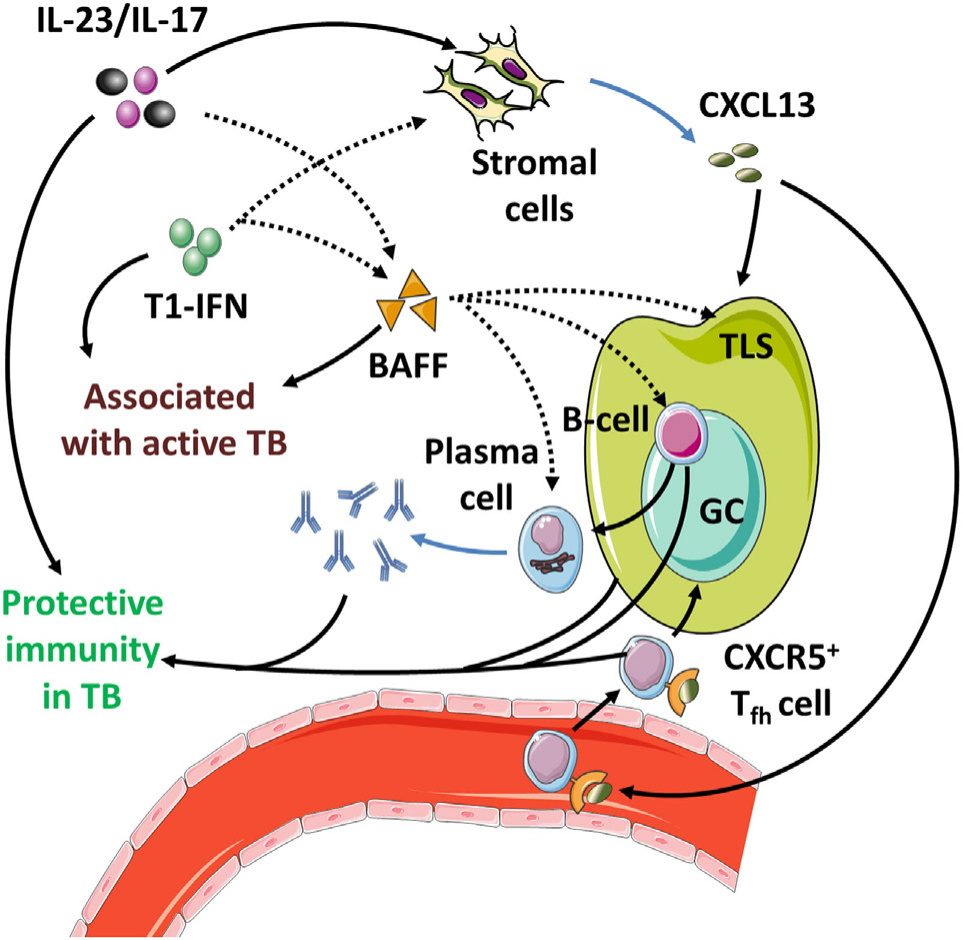
**Tertiary lymphoid structures (TLS) in tuberculosis (TB)**. TLS, Tfh cells, B-cells, and antibodies are all associated with protective immunity in TB. Preclinical TB models show that TLS induction and CXCL13 production are driven by IL-17 and IL-23. Type I interferons (T1-IFNs) and B-cell activating factor (BAFF) support TLS function and Tfh responses in autoimmune diseases (AID). In TB, T1-IFNs and BAFF are associated with active disease, but their functional role remains to be identified. Dotted lines implicate mechanisms present in AID that have not been confirmed in TB. Outstanding question: (1) What are the functional roles of T1-IFNs and BAFF in TLS function and humoral immunity in Mtb infection?

The newly appreciated functional roles of antibodies, B-cells, and T_fh_ cells in TB provide suggestive evidence that pathogenic mechanisms in AID confer protective immunity to TB. Further, insight into these mechanisms as discussed in Figures 6–8 may generate leads for immune-directed therapies adjunct to current and newly developed antimicrobial treatment protocols.

## Author Contributions

BM has written the manuscript and drafted the figures; EL has written the initial version of Section 4 and contributed to the conception of Figure 5; JS and TO have contributed substantially to Sections 1, 2, 3, and 5 and the conception of Figures 1–3; PL has contributed substantially to the design of the work, writing of the manuscript, conception of all figures, and final review of the manuscript. All authors have revised this manuscript for intellectual content, approved its final version for publication, and have agreed to be accountable for all aspects of the work and in ensuring that questions related to the accuracy or integrity of any part of the work are appropriately investigated and resolved.

## Conflict of Interest Statement

The authors declare that the research was conducted in the absence of any commercial or financial relationships that could be construed as a potential conflict of interest.
